# Vaccination herd effect experience in Latin America: a systematic literature review

**DOI:** 10.1080/21645515.2018.1514225

**Published:** 2018-09-19

**Authors:** Rodrigo DeAntonio, Sylvia Amador, Eveline M. Bunge, Jennifer Eeuwijk, David Prado-Cohrs, Javier Nieto Guevara, Maria del Pilar Rubio, Eduardo Ortega-Barria

**Affiliations:** a Centro de Vacunación Internacional S A CEVAXIN, Panama City, Panama; b GSK, Panama City, Panama; c Pallas Health Research and Consultancy BV, Rotterdam, the Netherlands; d GSK, Guatemala City, Guatemala; e GSK, Bogota Corporate Center, Bogotá, D.C., Colombia

**Keywords:** *Haemophilus influenzae* type b (Hib) vaccine, herd effect, Latin America, pneumococcal conjugate vaccine, rotavirus vaccine, systematic review

## Abstract

**Background**: National pediatric vaccination programs have been introduced in Latin America (LatAm) to reduce the burden of diseases due to pathogens such as rotavirus, *Haemophilus influenzae* type b (Hib) and pneumococcus. Vaccination health benefits may extend to unvaccinated populations by reducing pathogen transmission. Understanding herd effect is important for implementation and assessment of vaccination programs. The objective was to conduct a systematic review of published epidemiological evidence of herd effect with Hib, rotavirus and pneumococcal conjugate vaccines (PCV) in LatAm.

**Methods**: Searches were conducted in PubMed, Virtual Health Library (VHL), SciELO and SCOPUS databases, for studies reporting data on herd effect from Hib, rotavirus and PCV vaccination in LatAm, without age restriction. Searches were limited to articles published in English, Spanish or Portuguese (1990–2016). After screening and full-text review, articles meeting the selection criteria were included to be critically appraised following criteria for observational and interventional studies. The presence of a herd effect was defined as a significant decrease in incidence of disease, hospitalization, or mortality.

**Results**: 3,465 unique articles were identified, and 23 were included (Hib vaccine n = 5, PCV n = 8, rotavirus vaccine n = 10). Most studies included children and/or adolescents (age range varied between studies). Studies in adults, including older adults (aged > 65 years), were limited. Few studies reported statistically significant reductions in disease incidence in age groups not targeted for vaccination. Hib-confirmed meningitis hospitalization decreased in children but herd effect could not be quantified. Some evidence of herd effect was identified for PCV and rotavirus vaccine in unvaccinated children. Evidence for herd effects due to PCV in adults was limited.

**Conclusion**: After introduction of Hib, PCV and rotavirus vaccination in LatAm, reductions in morbidity/mortality have been reported in children not targeted for vaccination. However, due to methodological limitations (e.g. short post-vaccination periods and age range studied), there is currently insufficient evidence to quantify the herd effect in adult populations. More research and higher quality surveillance is needed to characterize herd effect of these vaccines in LatAm.

## Background

Latin America has introduced several new vaccines in recent years to reduce the burden of vaccine-preventable diseases,^1^ including vaccines against rotavirus, *Haemophilus influenzae* type b (Hib) and *Streptococcus pneumoniae* or pneumococcus. These pathogens represent a substantial burden of disease in Latin America. Rotavirus is a viral pathogen causing acute gastroenteritis leading to severe diarrhea, and mainly affects infants and young children.^2^ Diarrhea was the second most important cause of death worldwide in children aged 1–59 months in 2010, accounting for 4% of deaths in the Americas.^3^ In 2010, countries in the Americas were introducing rotavirus vaccine and were making efforts to increase vaccine coverage. Coverage ranged from 49% to 98% (median: 89%) in the 11 Latin America countries with vaccine introduction before 2010.^4^ *Haemophilus influenzae* (Hi) is a bacterium, commonly found in the nasopharynx of non-immune children. Hi disease is defined as invasive when the pathogen is found in normally sterile body fluids. There are six serotypes of Hi. Hib caused the majority of invasive disease before the introduction of the Hib vaccine. Hib can cause clinical disease including pneumonia and meningitis, with the greatest disease burden in children aged 4–18 months. In unvaccinated populations it is the dominant cause of non-epidemic bacterial meningitis in the first year of life. Even with prompt treatment, 3–20% of children with Hib meningitis die.^5^ Prior to vaccine introduction, approximately 20,000 cases of Hib meningitis were estimated to occur each year in Latin America and the Caribbean^6^ and approximately 33,000 cases of all Hib diseases (incidence rate 60 per 100,000).^7^ The Hib vaccine was targeted at the one Hib serotype that caused the majority of invasive Hi diseases. The pneumococcus bacterium is also commonly carried in the nasopharynx of young children. There are many different pneumococcal serotypes.^8^ It can cause invasive diseases such as pneumonia, meningitis and bacteremia, and has been estimated to cause 11% of all deaths in children aged 1–59 months worldwide.^9^ In developing countries, pneumococcal disease is estimated to result in over 800,000 deaths per year in children aged < 5 years.^10^ The pneumococcus is also a major cause of pneumonia in adults.^11^

Herd effect refers to a decreased disease incidence in unvaccinated segments of populations as a result of (typically) routine pediatric vaccination programs that reduce organism transmission within a population.^12^ Herd effect can include herd immunity and herd protection; of the two, herd protection is more clinically important.^13^ Herd immunity can occur in unimmunized individuals as a result of secondary exposure to attenuated virus/bacteria from the vaccine shed in fecal matter by immunized individuals.^13^ Herd protection can occur when vaccine coverage is sufficiently high to reduce pathogen transmission in the community, thereby reducing the risk of disease in both vaccinated and unvaccinated individuals.^13,14^ Some vaccines, e.g. rotavirus, can provide both herd protection and herd immunity, while others, e.g. pneumococcal vaccines, provide only herd protection.^13^ Herd protection can have important effects in populations who are not targeted for vaccination but are at high risk of infection; for example, routine pediatric pneumococcal vaccination has been shown to reduce invasive pneumococcal disease (IPD) in elderly adults (aged ≥ 65 years or more) who had not been vaccinated.^14^ Herd effect has reduced transmission of pertussis, protects against influenza and pneumococcal disease, and contributed to the eradication of smallpox.^12^ Moreover a recent study showed a 48.5% decline in the prevalence of Rotavirus (RV) in adults that coincides with similar declines observed in pediatric populations following widespread vaccination:^15^ these data strongly suggest an indirect effect of pediatric RV vaccination upon adult rotavirus disease. It is an important benefit of vaccination. Once a herd immunity threshold has been reached for a specific disease, herd immunity gradually eliminates the disease from the population.

Herd effect is assessed by measuring the change in disease incidence among the unvaccinated part of a partially vaccinated population over a given period of time, which may be manifested as a change in disease incidence in unvaccinated members of the vaccination cohort or in groups outside the vaccination cohort (e.g. unvaccinated age groups). The methods used to quantify changes in incidence vary by disease, and can include outcomes such as number of hospitalizations, number of physician visits or mortality rates. Herd effect is most frequently assessed using population surveillance studies before and after vaccination, cluster-randomized trials, and mathematical modelling.^14,16^ However, herd effect is complex and studies, particularly before-and-after studies, can be confounded by many factors. These may include secular trends in incidence, differences in pre-vaccine serotype distribution, under-reporting, socioeconomic factors such as living conditions, genetic or medical predisposing factors, changes in antibiotic use/prescribing practices, variations in behavioral factors such as attendance at school or day care, changes in surveillance system, access to health services and health-seeking behaviors.^8,16,17^ Before-and-after studies of vaccine impact are thus difficult to interpret.^8^ Adjustments should be made for factors such as the effectiveness and duration of vaccine-induced protection, rebound effects such as serotype replacement and behavioral changes.^14^ Herd effect may also vary from one geographical region to another, reflecting differences in factors such as the age of peak disease risk (e.g. peak risk for pneumococcal disease appears to be earlier in infancy in developing countries, compared with the USA in the era before pneumococcal conjugate vaccines [PCV]), and vaccination schedules^18^ Other factors that might influence herd effect are population density and vaccine uptake. Extrapolation of herd effect from one geographical area to another, such as extrapolation from studies in Europe to Latin America, should be avoided.

The vaccines against rotavirus, Hib and pneumococcus in Latin America have already been shown to have a positive health impact in targeted groups.^6,19–23^ Several studies have reported evidence of herd protection with these vaccines in a range of countries.^24,25^ As herd effect is a valuable extended benefit of vaccination, it is important to consider the available evidence on herd effect as well as the effects on targeted groups. The objective of the present systematic review was to summarize all the available published epidemiological evidence of herd protection with Hib, rotavirus and PCV in all age groups in Latin America and the Caribbean. It will be a valuable tool for healthcare decision-makers and public health authorities assessing vaccination programs in the region.

## Results

### Search results

**Figure 1.**
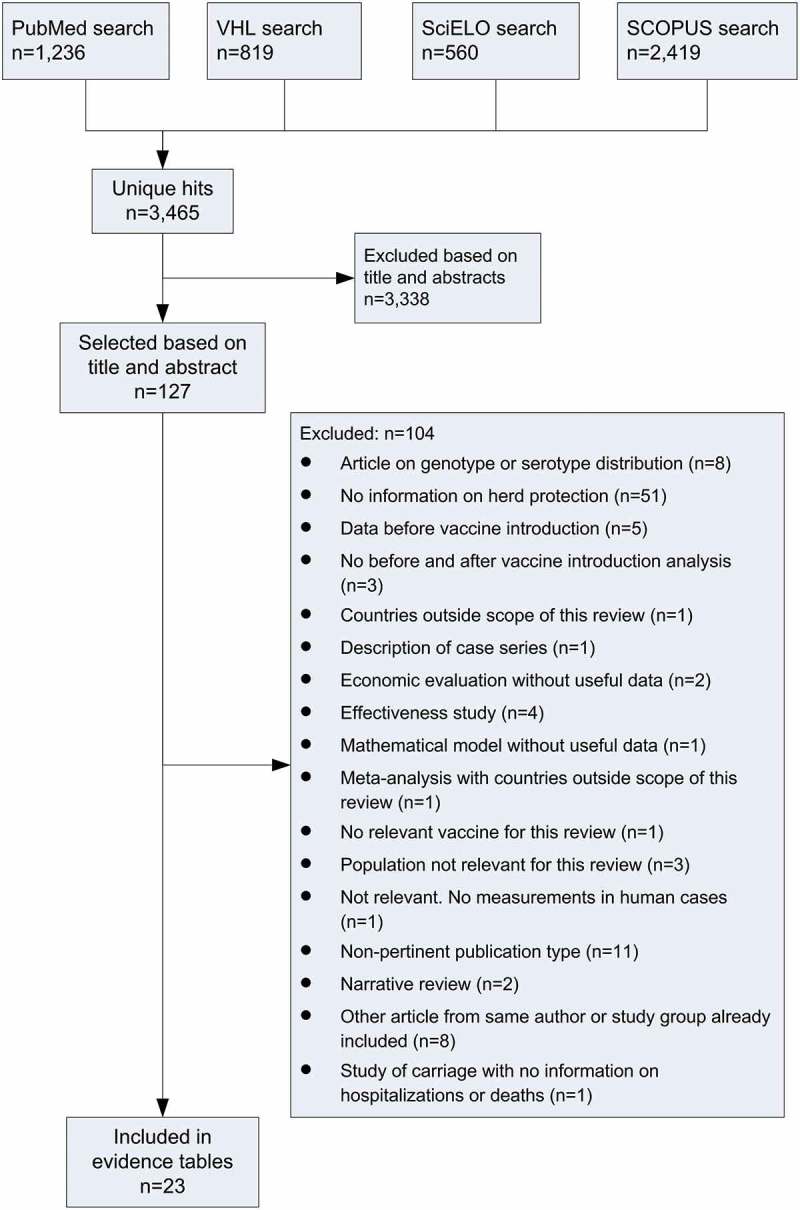
Search results and study selection.

After removal of duplicates, 3,465 articles were identified from the literature searches, and 127 were obtained for full-text review. Of these, 23 were included in the review (Figure 1). Eight publications were not included because other articles from the same author or study group analyzing the same data were already included.

Five studies reported data on Hib vaccine,^26–30^ eight on pneumococcal vaccine,^31–38^ and the remaining ten studies reported data on rotavirus vaccine.^39–48^ The studies came from nine countries, Argentina, Brazil, Chile, Cuba, El Salvador, Mexico, Nicaragua, Panama, and Uruguay.

### Hib vaccine

#### Hib invasive disease

One study, conducted in Uruguay, presented data on the incidence of Hib invasive disease (Table 1).^29^ After vaccine introduction in 1994, the incidence of invasive disease in children aged < 36 months declined from 31 cases per year in 1993 and 34 cases per year in 1994 to only one case per year from 1996 onwards, so although the large decline could indicate a possible herd effect, this could not be quantified.

Four studies described the incidence of hospitalization for Hib-confirmed meningitis after introduction of Hib vaccination, three in Brazil^27,28,30^ and one in Cuba^26^ (Table 1). All four studies showed a decrease in incidence after vaccination in children aged < 1 year, the age group targeted for vaccination (Supplementary Table 1). In children aged < 1 year in Brazil incidence decreased from 36.5 per 100,000 to 3.4 per 100,000 (p < 0.005), and in children aged 1–4 years the decrease was from 6.4 per 100,000 to 0.7 per 100,000 (p < 0.02).^27^ All four studies included older children, whereas only one^27^ included adults, with a very low incidence previous to vaccine introduction; this study has a post-vaccination period of 2 years (Table 1).

**Table 1.** Studies reporting data on Hib vaccine and possible demonstration of herd effects in Latin America in groups not targeted for vaccination.

Reference Country	Vaccine type Schedule and age groups Year of vaccine introduction	Coverage and year coverage was assessed	# years before vaccination	# years after vaccination	Subgroups	Change in incidence	Herd protection effect
**Hospitalization for Hib-confirmed meningitis**							
**Miranzi, 2006 [28]** Brazil (national)	Hib CRM197 conjugated vaccine Infant < 1 year of age Vaccine introduction in 1999	2000: 92%	16 (1983–1999)	3 (2000–2002)	**Age groups**		
					5–9 yrs	−49,6%^1^	Age group not targeted for vaccination
					≥ 10 yrs	−57,3%^1^	Age group not targeted for vaccination
**Ribeiro, 2007 [30]** Brazil (regional)	Hib conjugate vaccine 3 doses: at 2, 4 and 6 months of age Catch up: During the 1st and 2nd year of the Hib immunization campaign, children 12–23 months of age received a single dose of the vaccine Vaccine introduction in August 1999	2000: 82% 2001: 77% 2002: 69% 2003: 86% 2004: 82% Catch up	3 (August 1996 – August 1999)	5 (August 1999 – August 2004)	**Age groups**		
		1999: 37%			3–4 yrs	−67,5%	Age group not targeted for vaccination ^3^
		2000: 24%			5–9 yrs	−26,6%	Age group not targeted for vaccination ^3^
**Kmetzsch, 2003 [27]** Brazil (regional)	Conjugate Hib vaccine The Hib vaccine was implemented for children under 2 Vaccine introduction in 1999. 1999 can be considered a transitional year with low coverage	2000: 88% 2001: 87%	1 (1998)	2 (2000–2001)	**Age groups**		
					5-< 10 yrs	−25,0%	Age group not targeted for vaccination ^5^
					10-< 15 yrs	−50,0%	Age group not targeted for vaccination ^5^
					15-< 20 yrs	−50,0%	Age group not targeted for vaccination ^5^
					≥ 20 yrs	−^4^	Age group not targeted for vaccination ^5^
**Dickinson Meneses, 2002 [26]** Cuba (national)	Haemophilus influenzae type b (Hib) vaccine Infants of 2, 4 and 6 months old were vaccinated Booster was given at 18 months Vaccine introduction in 1999.	1999: 100% 2000: 78%	1 (1998)	1 (2000)	**Age groups**		
					5–9 yrs	−77,8%	Age group not targeted for vaccination ^6^
					10–14 yrs	−100,0%	Age group not targeted for vaccination ^6^
**Mortality from Hib-confirmed meningitis**							
**Miranzi, 2006 [28]** Brazil (national)	Hib CRM197 conjugated vaccine Infant < 1 year of age Vaccine introduction in 1999	2000: 92%	16 (1983–1999)	3 (2000–2002)	**Age groups**		
					5–9 yrs	−50,0%^1^	Age group not targeted for vaccination ^7^
					≥ 10 yrs	−52,4%^1^	Age group not targeted for vaccination ^7^
**Invasive Hib disease**							
**Montano, 2001 [29]** Uruguay (national)	*H. influenzae* type b conjugate-vaccine, polyribosyl-ribitol-phosphate conjugated to tetanus toxoid In infants younger than 7 months, three doses (every two months) and a booster dose at 12 months of age; between 7 and 11 months, two doses with a booster between 12 and 15 months; between 1 and 4 years, a single dose Vaccine introduction in 1994. 1995 was considered as the year when children < 1 years should have received two doses and 1–5 year olds should have received one dose.	76% in children aged 2 months to 4 years, during period 29 August 1994 to 31 December 1994	2 (1993–1994)	5 (1995–1999)	< 36 months	−94.5%	Age group not targeted for vaccination ^8^

Hib: *Haemophilus influenzae* type b; yr(s): year(s)

1: Pre-vaccination mean incidence was calculated using the last five years before vaccine introduction (1995–1999);

2: Some of the children in this age group might have received the vaccine. Change in incidence is likely caused by a combination of vaccine effect and herd protection;

3: During the last year of surveillance no cases of Hib meningitis were observed. The low pre-vaccine incidence does not allow to quantify herd protection effects;

4: Hospitalization incidence rate was very low in adults ≥ 20 years old. In 1998 incidence was 0 per 100,000 individuals, 0.08 in 1999, 0 in 2000, 0.08 in 2001;

5: Hospitalization incidence rates were already very low before introduction of the vaccine and remained low. The low pre-vaccine incidence does not allow to quantify herd protection effects;

6: Before introduction of the vaccine, the incidence of total bacterial meningitis and bacterial meningitis caused by Hib was already low in children 5–9 and 10–14 years old. After vaccination the Hib meningitis incidence decreased to zero occurrence of Hib meningitis. The low pre-vaccine incidence does not allow to quantify herd protection effects;

7: Mortality rates were already very low in these age groups before introduction of the vaccine. In 2002 no mortality from Hib meningitis was registered in 5–9 year olds and ≥ 10 year olds. the low pre-vaccine incidence does not allow to quantify herd protection effects;

8: From 1996 to 1999 only one case per year of invasive disease was reported, so although the large decline could indicate a possible herd effect, this could not be quantified.

In a national study in Brazil,^28^ the incidence of hospitalization for Hib-confirmed meningitis in children aged 5 years or older, who were not in the age group targeted for vaccination, decreased by 49.6–57.3% in the three years after vaccine introduction compared with the 16 years before vaccine introduction (Table 1). In the last year of the study (2002), hospitalization incidence was 0.13 per 100,000 in children aged 5–9 years (compared with 0.23 in 1983) and 0.02 per 100,000 in children aged ≥ 10 years (compared with 0.04 in 1983). In the Metropolitan Salvador region of Brazil, where a program of Hib vaccination in infants with a catch-up campaign in children aged 12–23 months was introduced, hospitalization for Hib-confirmed meningitis fell by 67.5% in children aged 3–4 years and by 26.6% in children aged 5–9 years^30^ (Table 1). However, the incidence was already low before vaccination (7.87 per 100,000 in children aged 3–4 years and 1.26 per 100,000 in children aged 5–9 years in the last year before vaccination), and the 95% confidence interval (CI) included zero in all except two of the post-vaccination years in the group aged 3–4 years, so it was difficult to quantify any herd effect. In the region of Rio Grande do Sul, the incidence of Hib-confirmed meningitis was already very low before vaccine introduction (0.4 per 100,000 in children aged 5–10 years, 0.1 per 100,000 in age 10–15 and age 15–20 years, and 0.0 per 100,000 in adults aged ≥ 20 years) and remained low (0.2 per 100,000, 0.0 per 100,000, 0.1 per 100,000 and 0.08 per 100,000, respectively, two years after vaccine introduction), so it was not possible to detect or quantify any herd protection effect^27^ (Table 1).

The study in Cuba reported decreases in the incidence of hospitalization for Hib-confirmed meningitis in all age groups, including age groups not targeted for vaccination (Table 1). The incidence of hospitalization for Hib meningitis in individuals aged 5–14 years was low before vaccine introduction (0.9 per 100,000 in the group aged 5–9 years and 0.3 per 100,000 in the group aged 10–14 years in 1998) and decreased to almost zero after vaccination (0.2 per 100,000 and 0 per 100,000, respectively, in 2000).^26^ Herd protection may have contributed to this very low incidence, but it is not possible to quantify any effect.

Incidence in populations not targeted for vaccination was very low in all four studies, which limits the possibility to draw any conclusions from these studies.

#### Hib-confirmed meningitis mortality

A national study in Brazil reported that the incidence of mortality from Hib-confirmed meningitis fell by over 80% in children aged < 1 year (the group targeted for vaccination, Supplementary Table 1) and by half after vaccine introduction in children aged 5 years or older, who were not in the age group targeted for vaccination, indicating a possible herd protection effect (Table 1).^28^ However, Hib-meningitis mortality was low in children aged 5 years or older (0.05 per 100,000 in children aged 5–9 years and 0.0 per 100,000 in children aged ≥ 10 years in 1983, falling to 0.0 per 100,000 in both groups in 2002), so any herd effect was difficult to quantify (Table 1).

### Pneumococcal conjugate vaccines (PCV)

#### Invasive pneumococcal disease (IPD)

Four studies investigated changes in the incidence of IPD after introduction of pneumococcal vaccination, two in Brazil,^31,33^ one in Chile^38^ and one in Uruguay^34^ (Table 2 and Supplementary Table 2). One of the studies in Brazil [32] contains both data on overall IPD and vaccine-type IPD, these two datasets have been entered separately in Table 2 and Supplementary Table 2 and are discussed separately below.

**Table 2.** Studies reporting data on possible herd effects against invasive pneumococcal disease and pneumococcal pneumonia after introduction of pneumococcal conjugate vaccine in Latin America in groups not targeted for vaccination.

References Country	Vaccine type Schedule and age groups Year of vaccine introduction	Coverage and year coverage was assessed	# years before vaccination	# years after vaccination	Subgroups	Change in incidence	Evidence of herd effect
**Overall IPD**							
**Andrade, 2016 [31]** Brazil (national)	10-valent pneumococcal vaccine (PHID-CV) Vaccination at 2, 4, and 6 months plus a booster at 12 to 15 months of age Catch-up schedule for children aged 7–11 months (two doses plus booster) and 12–23 months (single catch-up dose) during first year of vaccine introduction Vaccine introduction in 2010, therefore 2010 is considered a transitional year	2011: 82% 2012: 88% 2013: 92%	2 (2008–2009)	3 (2011–2013)	**Age groups** **PHID-CV types/total cases^1^**		
					5–9 yrs	−13.1	Age group not targeted for vaccination
					10–17 yrs	−35.4	Age group not targeted for vaccination
					18–39 yrs	−21.1*	Age group not targeted for vaccination ^2^
					40–64 yrs	−39.5*	Age group not targeted for vaccination ^2^
					≥ 65 yrs	−23.2*	Age group not targeted for vaccination ^2^
**dos Santos, 2013 [33]** Brazil (regional)	PHID-CV 3 doses in the first 6 months, with a booster dose at 12–15 months of age Vaccine introduction in 2010	NR	4.5 (Jan 2006- June 2010)	2 (July 2010- Sept 2012	**Age groups** **All serotypes**		
					2-< 15 yrs	−24.8%	Age group not targeted for vaccination ^3^
					≥ 15 yrs	83.7%	Age group not targeted for vaccination
**Valenzuela, 2014 [38]** Chile (national)	PHID-CV Vaccination started in 2011 with a 4-dose schedule at 2, 4, 6 and 12 months of age. In 2012 the schedule changed to 3 doses at 2, 4 and 12 months of age. Vaccine introduction in 2011	2011: 55% 2012: 82%^4^	3 (2007–2010)	1^5^ (2012)	**Age groups** **All serotypes**		
					12–23 mo	−53.2%***	Age group not targeted for vaccination
					24–59 mo	−5.3%	Age group not targeted for vaccination ^6^
					5–64 yrs	−6.3%	Age group not targeted for vaccination ^6^
					≥ 65 yrs	13.7%	Age group not targeted for vaccination ^6^
**Garcia Gabarrot, 2014 [34]** Uruguay (national)	PCV7, PVC13 PCV7 (March 2008) 2 + 1 schedule (doses administered at 2, 4 and 12 months of age). During the first year, a 2-dose catch-up program was also offered to children up to 2 years old. PCV13 (March 2010) same 2 + 1 schedule was used. Children with 1 or 2 doses of PCV7 completed their schedule with PCV13. Additionally, a single dose catch-up of PCV13 was offered to all children born between 2005 and 2008. Vaccine introduction: PCV7 March 2008 and PCV13 March 2010	2010: 96.9–98.9%^7^	5 (2003–2007)	4^8^ (2009–2012)	**Age groups** **All serotypes**		
					2–4 yrs	−54,1%*	Age group not targeted for vaccination ^9^
					5–14 yrs	−45,9%	Age group not targeted for vaccination ^10^
					15–59 yrs	102,9%	Age group not targeted for vaccination ^11^
					> 60 yrs	270,9%*	Age group not targeted for vaccination ^11^
**Vaccine-type IPD**							
**dos Santos, 2013 [33]** Brazil (regional)	PHID-CV 3 doses in the first 6 months, with a booster dose at 12–15 months of age Vaccine introduction in 2010	NR	4.5 (Jan 2006- June 2010)	2 (July 2010- Sept 2012)	**Age groups** **PHID-CV serotypes**		
					2-< 15 yrs	−65.5%	Age group not targeted for vaccination ^3^
					≥ 15 yrs	32.9%	Age group not targeted for vaccination
**Garcia Gabarrot, 2014 [34]** Uruguay (national)	PCV7, PVC13 PCV7 (March 2008) 2 + 1 schedule (doses administered at 2, 4 and 12 months of age). During the first year, a 2 dose catch-up program was also offered to children up to 2 years old. PCV13 (March 2010) same 2 + 1 schedule was used. Children with 1 or 2 doses of PCV7 completed their schedule with PCV13. Additionally, a single dose catch-up of PCV13 was offered to all children born between 2005 and 2008. Vaccine introduction: PCV7 March 2008 and PCV13 March 2010	2010: 96.9–98.9%^7^	5 (2003–2007)	4^8^ (2009–2012)	**Age groups** **PCV7 serotypes**		
					5–14 yrs	−63,6%	Age group not targeted for vaccination ^10^
					15–59 yrs	42,1%	Age group not targeted for vaccination ^11^
					> 60 yrs	206,2%	Age group not targeted for vaccination ^11^
					**PCV13 serotypes**		
					5–14 yrs	−50,7%	Age group not targeted for vaccination ^10^
					15–59 yrs	52,7%	Age group not targeted for vaccination ^11^
					> 60 yrs	195,6%	Age group not targeted for vaccination ^11^
**Pneumococcal pneumonia**							
**Pirez, 2014 [37]** Uruguay (regional)	7-valent pneumococcal conjugate vaccine (PCV7)/13-valent PCV (PCV13) PCV7: 2 + 1 schedule (given at 2, 4 and 12 months of age). Catch-up immunization was offered to children born in 2007 (2 doses, at 15 and 17 months of age) Uruguay switched to 13-valent PCV (PCV13) with same vaccination schedule in April 2010. Catch-up immunization was offered to children born from January 1, 2005 to April 23, 2009, with a single dose of PCV13. Vaccine introduction: PCV7 in March 2008, replaced by PCV13 in April 2010	National vaccination data demonstrated high compliance with PCV7/13 use: ≥ 93% of children received 3 doses (cohort 2008 and 2009) and 98% and 95% have been vaccinated with 1 and 2 doses of PCV13, respectively, for cohort 2010	5 (2003–2007)	5 (2008–2012)	**P-CAP** **0–14 yrs**		
					**P-CAP PCV7 serotypes** **0–14 yrs**	−79.4%^13^	Age group not targeted for vaccination ^15^
					**P-CAP PCV13 serotypes** **0–14 yrs**	−60.2%^14^	Age group not targeted for vaccination ^15^

PCV-7: PCV-7-valent; PCV-13: PCV-13-valent; P-CAP: community-acquired pneumonia caused by *S. pneumoniae*; PCV: pneumococcal conjugate vaccine; PHiD-CV: 10-valent pneumococcal vaccine; mo: months; yr: year; yrs: years.

Incidence in studies presented as: per 1,000 population individuals (dos Santos et al.[32]), per 100,000 individuals population (Garcia Gabarrot et al.[33]) and cases per year (Valenzuela et al. [37])

*A significant reduction (p < 0.05) in incidence per 1,000 population individuals was observed.

**A significant reduction (p < 0.01) in incidence per 1,000 population individuals was observed.

*** Odds ratios for 12 months 2007–2010 vs. 2011 and 2007–2010 vs. 2012, 12–23 months 2007–2010 vs. 2012, 5–64 years 2007–2010 vs. 2011 showed a significant reduction after vaccination.

1: Cases of IPD caused by PHiD-CV serotypes as percentage of total reported cases of invasive pneumococcal disease;

2: Overall number of cases of invasive pneumococcal disease increased in the period after introduction of the vaccine. Incidence of invasive pneumococcal disease caused by PHiD-CV serotypes increased in adults (> 18 yrs);

3: Decline in the age group 2-< 15 years was not statistically significant. The incidence dropped from 3.35 to 2.52 per 1,000 individuals in all serotypes and 2.81 to 0.97 per 1.,000 individuals in PHiD-CV serotypes, which is larger than can be explained by only environmental factors. However power of the study might be too low to find a significant effect in this age group.

4: According to the PAHO website;

5: Vaccine introduction in 2011, therefore 2011 is considered a transitional year and excluded from analysis;

6: The decrease is small and only borderline significant in the first year after introduction, but not significant in the second year after introduction. In the 24–59 months there is no significant decline in both years. Small declines could also be caused by other factors, such as seasons, changes in health care, etc.;

7: Among new cohorts;

8: Vaccine introduction in 2008, therefore 2008 is considered a transitional year and excluded from analysis;

9: There is a decline in this age group, but only after 2009, which might indicate this decline is caused by immunity gained by immunization in 2008. Between 2008 and 2009 there was an increase in incidence of IPD;

10: The incidence of IPD declined in PCV-7 and PCV-13 types, but increased in not- PCV- types;

11: The incidence of IPD increased in 15–59 year olds and ≥ 60 year olds.

12: In 2012 seven cases of pneumonia caused by *S. pneumoniae* were registered;

13: In 2011 and 2012 no cases of pneumonia caused by a serotype of *S. pneumoniae* included in PCV7 were registered;

14: In 2012 two cases of pneumonia caused by a serotype of *S. pneumoniae* included in PCV13 were registered;

15: Since children aged 0 to 14 years are included in one group, it is not possible to separate the effect in age groups targeted for vaccination and not targeted for vaccination. In 2011 and 2012 no cases of P-CAP PCV-7 serotypes were observed, suggesting herd protection. Cases caused by PCV-13 decline to two cases in 2012.

All four studies showed a reduction in the incidence of overall IPD^31,33,34,38^ in the age group targeted for vaccination (Supplementary Table 2).

In Brazil, the observed rate of overall IPD was 2.1 per 100,000 in children aged 5–9 years,^31^ 4.7% lower than the predicted rate of 2.2 per 100,000 although the difference was not statistically significant (p = 0.660). In the other age groups not targeted for vaccination, the observed rates were higher than the predicted rate (p < 0.05 in age groups 18–39 years, 40–64 years and ≥ 65 years) (Table 2). The observed and predicted rates could be biased by the introduction of an enhanced surveillance of IPD that was put in place all over the country in order to increase case detection for a case control study to assess PCV-10 initiated shortly after PCV-10 introduction. This surveillance was likely to affect the number of cases registered with IPD.

In another study in Brazil,^33^ the overall IPD incidence pre-vaccination was 3.35 per 1,000 in the group aged 2–15 years and 1.72 per 1,000 in the group aged ≥ 15 years, and these did not significantly change post-vaccination (2.52 per 1,000 and 3.16 per 1,000, respectively, p = not significant) (Table 2). In the age group targeted for vaccination (aged < 2 years), incidence of overall IPD decreased from 20.30 per 1,000 pre-vaccination to 3.97 per 1,000 post-vaccination (p < 0.0012) (Supplementary Table 2). Both Brazilian studies had a post-vaccination follow-up of 3^31^ and 2 years,^33^ respectively.

In Chile, the incidence of overall laboratory-confirmed IPD decreased from 56.1 per 100,000 in 2007 (prior to vaccination, which began in 2011) to 16.3 per 100,000 in 2012 in children aged < 12 months.^38^ There was also a decrease from 42.0 to 19.9 per 100,000 in children aged 12–23 months. No decline was seen in groups aged 24–59 months, 5–64 years or ≥ 65 years.^38^

In Uruguay,^34^ the incidence of overall IPD decreased significantly (p < 0.01) in children aged < 2 years (Supplementary Table 2). In children aged 2–4 years, who were not targeted for vaccination, overall IPD incidence decreased from 23.82 per 100,000 pre-vaccination to 10.93 per 100,000 post-vaccination (p = 0.0279) (Table 2). In the groups aged 5–14 years and 15–59 years there was no significant changes in overall IPD incidence. In the group aged ≥ 60 years there was a significant increase in overall IPD incidence (from 3.23 per 100,000 to 11.98 per 100,000, p = 0.0461)^34^ (Table 2). This study had a post-vaccination period of 4 years.

#### Vaccine-type IPD

Two studies reported data on vaccine-type IPD, and both showed a reduction in the incidence of vaccine-type IPD^33,34^ in the age group targeted for vaccination (Supplementary Table 2).

In a study in Brazil^33^ the incidence of vaccine-type IPD pre-vaccination in the groups aged 2–15 years and ≥ 15 years was 2.81 per 100,000 and 0.85 per 100,000, respectively, and the incidence post vaccination was 0.97 per 100,000 and 1.13 per 100,000, respectively (p = not significant). In the age group targeted for vaccination (aged < 2 years), incidence of vaccine-type IPD decreased from 16.47 per 100,000 to 0.44 per 100,000 (p = 0.0002) (Table 2).

In Uruguay,^34^ the incidence of vaccine-type IPD decreased significantly (p < 0.01) in children aged < 2 years (Supplementary Table 2). In children aged 2–4 years, who were not targeted for vaccination, PCV-7 vaccine-type IPD incidence decreased from 6.97 per 100,000 to 1.73 per 100,000 (p = 0.0971), and PCV-13 vaccine-type IPD incidence decreased from 16.05 per 100,000 to 7.29 per 100,000 (p = 0.0681). In the groups aged 5–14 years, 15–59 years and ≥ 60 years there was no significant changes in vaccine-type IPD incidence (Table 2).

#### Pneumococcal pneumonia

One study reported data on the incidence of community-acquired pneumonia caused by serotypes included in PCV-7 or PCV-13 in Uruguay^37^ (Table 2). Uruguay introduced vaccination with PCV-7 in March 2008 and switched to PCV-13 in April 2010. In both cases a catch-up program was implemented. This study included children aged 0–14 years, and did not distinguish between age groups targeted or not targeted for vaccination. Although the publication did not meet our formal inclusion criteria, information was available from another published study^49^ that allowed us to separate target and non-target age groups in this study. The study therefore met our inclusion criteria when both publications were considered together. Cases of community-acquired pneumonia caused by *Streptococcus pneumoniae* decreased from an incidence of 62 per 10,000 discharges in 2003 to 6.4 per 10,000 discharges in 2012. The incidence of PCV-7 vaccine-type pneumonia decreased from 36 per 10,000 discharges in 2003 to zero cases in 2011 and 2012. The incidence of PCV-13 vaccine-type pneumonia decreased from 23 cases per 10,000 discharges in 2003 to 2 cases per 10,000 discharges in 2012^37^ (Table 2). The decrease of PCV-7 types to zero indicated that the disease was also absent in non-targeted and/or non-vaccinated children in the age group, which could indicate a herd effect.

#### All-cause pneumonia

The review identified three studies on the incidence of all-cause pneumonia, conducted in Argentina,^35^ Nicaragua^32^ and Uruguay.^36^ All three studies showed a reduction in incidence in the age group targeted for vaccination (Supplementary Table 3).

**Table 3.** Studies reporting data on possible herd effects against all-cause pneumonia after introduction of pneumococcal conjugate vaccine in Latin America in groups not targeted for vaccination.

Reference Country	Vaccine type Schedule and age groups Year of vaccine introduction	Coverage and year coverage was assessed	# years before vaccination	# years after vaccination	Subgroups	Change in incidence	Herd protection effect
**Gentile, 2015 [35]** Argentina (regional)	13-valent pneumococcal conjugate vaccine (PCV13) A “2 + 1” schedule (one dose at 2 months old, another dose at 4 months old, and a booster dose at 1 year old). During the first year after introducing the vaccine, children aged between 12 and 24 months old were also immunized with two doses. Vaccine introduction in January 2012	2012 1st dose: 100% 2nd dose: 83% 3rd dose: 48.3% 2013 1st dose: 87.6% 2nd dose: 84.9% 3rd dose: 61.3%	3 (2003–2005)	2 (2012–2013)	**Age groups**		
					24–59 mo	−20,7%	Age group not targeted for vaccination ^1^
**Becker-Dreps, 2014 [32]** Nicaragua (regional)	13-valent pneumococcal vaccine (PCV-13) A “3 + 0” dosing schedule, at 2, 4 and 6 months of age. During the first year of the immunization program, a single catch-up dose was also provided to children aged 12–24 months. Vaccine introduction in 2010	2011: < 1 yr: 63%^2^ (range by municipality: 49–71%); 1-< 2 yr: 87%^3^ (range by municipality: 68–100%) 2012 < 1 yr: 97%^2^ (range by municipality: 80–100%); 1-< 2 yr: 98%^3^ (range by municipality: 80–100%)	3 (2008–2010)	2 (2011–2012)	**Incidence rate ratio** **Age groups** **Hospitalization**		
					24–59 mo	0.73	Age group not targeted for vaccination ^4^
					5–14 yrs	0.81	Age group not targeted for vaccination ^4^
					**Ambulatory visits for pneumonia**		
					24–59 mo	0.92	Age group not targeted for vaccination ^4^
					5–14 yrs	0.95	Age group not targeted for vaccination ^4^
**Hortal, 2014 [36]** Uruguay (regional)	Pneumococcal conjugate vaccine, 7-valent and 13-valent PCV7 (2008): a 2 + 1 dosing schedule (2, 4 and 12 months of age) and a two doses catch-up, was offered to the 2007 cohort at 15 and 17 months of age. PCV13 (2010): same dosing schedule, and a catch-up was offered to children up to 5 years of age. Vaccine introduction in 2008, with a replacement of vaccine type in 2010. 2009–2012 were considered post-vaccine follow-up years.	At least one vaccine dose, by the end of 2012, was 97.7% for PCV7 and 99.8% for PCV13	5 (2001–2004)	3 (2009–2012)	**Age groups**		
					48–59 mo	−1,6%	Age group not targeted for vaccination

PCV-7: PCV-7-valent; PCV-13: PCV-13-valent; CI: confidence interval; mo: months; PCV: pneumococcal conjugate vaccine; yr: year; yrs: years

Change in incidence calculated for Gentile and Hortal. Gentile [34] presented incidence rate per 100,000 individuals, Hortal [35] presented consolidated pneumonia incidence per 100,000 person-years; Becker-Dreps [31] calculated incidence rate ratios.

1: There is a small decline of incidence in this age group, however it is not significant and in 2013 the incidence increased. There might be a small effect of herd protection, but natural fluctuations in incidence or effects of environmental factors cannot be ruled out;

2: Received all three doses of the vaccine;

3: Received one dose;

4: The incidence rate ratios of 24-to-50-month-old and 5-to-14-year-old children showed there is a significant decline. However there might be effects of natural fluctuations in incidence or environmental factors that cannot be ruled out.

These studies suggested little or no detectable herd protection effect against all-cause pneumonia in children not targeted for vaccination (Table 3). In Argentina, there was little change in the incidence of all-cause pneumonia (excluding nosocomial pneumonia) in children aged 24–59 months (not targeted for vaccination). The incidence was 321 per 100,000 pre-vaccination and 260 per 100,000 in 2013 (vaccine effectiveness 18.8%, not statistically significant),^35^ indicating little or no detectable herd protection effect. In Nicaragua, the incidence of hospitalization for all-cause pneumonia decreased after vaccine introduction in children aged 5–14 years who were not targeted for vaccination (incidence rate ratio 0.81, 95% CI 0.72, 0.90).^32^ A small herd protection effect could be possible. In Uruguay, there was a reduction in consolidated pneumonia hospitalizations in children aged 36–59 months from 741 per 100,000 to 633 per 100,000 that did not reach statistical significance.^36^ Additional data from the same study reported no decrease in hospitalizations in children aged 5–14 years, suggesting no herd protection effect in this age group.

No studies reporting data on possible herd effect for IPD and mortality were identified.

### Rotavirus

#### Hospitalizations for rotavirus-associated gastroenteritis or diarrhea

Three studies reported data on the incidence of hospitalizations for rotavirus-associated gastroenteritis or diarrhea, two from Brazil^42,47^ and one from El Salvador.^48^ All three studies reported a reduction in incidence in age groups targeted for vaccination (Supplementary Table 4), of 82.1% in children aged < 1 year in Brazil,^47^ and 81.5% (95% CI 74, 83) two years after vaccination in children aged < 1 year in El Salvador.^48^ In children aged < 5 years in El Salvador, the reduction in hospitalization was 81% in 2008 (p < 0.0001) and 69% in 2009 (p < 0.0001).^48^

**Table 4.** Studies reporting data on potential herd effects against hospitalizations for rotavirus gastroenteritis and diarrhea after introduction of rotavirus vaccine in Latin America in groups not targeted for vaccination.

References Country	Vaccine type Schedule and age groups Year of vaccine introduction	Coverage and year coverage was assessed	# years before vaccination	# years after vaccination	Subgroups	Change in incidence	Herd protection effect
**Gurgel, 2014 [42]** Brazil (national)	Monovalent G1P[8] Rotarix vaccine Two doses to all children < 3 months of age Vaccine introduction at the end of 2006	2006: 28.8% of the patients were vaccinated 2012: 86.7% of the patients were vaccinated	8 (1998–2005)	6 (2007–2012)	Data were presented in figures. Age groups were: -< 1 year 1-< 2 years ≥ 2 years		The vaccine was associated with reduction in the proportion of children attending the hospital, hospitalizations caused by rotavirus, in all age groups
**Sáfadi, 2010 [47]** Brazil (regional)	Rotavirus vaccine (Rotarix) Two doses of rotavirus vaccine before the age of 12 months Vaccine introduction in March 2006. In this study the year 2006 was considered a transition year.	2006: 54% 2007: 78% 2008: 81%	2 (2004–2005)	2 (2007–2008)	**Age groups**		
					2-< 5 yrs	29.4%	Age group not targeted for vaccination ^1^
**Yen, 2011 [48]** El Salvador (national)	Rotavirus vaccine Rotavirus vaccination is recommended for administration in 2 doses at 2 and 4 months of age. Vaccine introduction in 2006. 2007 was considered a transitional year during which rotavirus vaccine was still being introduced.	2008, first dose < 1 yr: 76% 1-< 2 yr: 84% ≥ 2 yrs not vaccinated 2009, first dose	1 (2006)	2 (2008–2009)	**Age groups**		
					3–4 yrs	26,7%	Age group not targeted for vaccination ^2^
					4-< 5 yrs	−40,4%	Age group not targeted for vaccination ^2^

mo: months; yr: year; yrs: years

1: There is a decline in incidence during the transition year, however in the two years after introduction (2007–2008) there is a small increase, but post-vaccine incidence was below pre-vaccine incidence;

2: The years after immunization, 2008 and 2009, did not show a similar trend. In 2008 a decline is seen in age groups not vaccinated, which could suggest herd protection effect. However, this effect is not seen in 2009. In 2009 an increase was seen in rotavirus hospitalizations in children 3 to < 5 years old, suggesting no herd protection effect in 2009, while the number of cases in other age groups further declined.

In a study at a hospital in Sao Paulo, Brazil,^47^ hospitalizations due to rotavirus-associated gastroenteritis decreased by 29.4% in children aged 24–59 months, who were not in the age group targeted for vaccination, and the rotavirus positivity rate decreased by 23.9% (from 35.0% to 26.6%) although the decline was not statistically significant (p = 0.2)^47^ (Table 4). A national study in Brazil^42^ reported a reduction in the proportion of diarrhea cases positive for rotavirus, although there were large variations between the years and conclusions cannot easily be drawn. Rotavirus-related hospitalizations in El Salvador^48^ decreased in age groups not targeted for vaccination (children aged ≥ 2 years) in 2008, from 123 per 100,000 to 43 per 100,000 (65% reduction, 95% CI 50, 75) in children aged 2–3 years, from 30 per 100,000 to 18 per 100,000 (41% reduction, 95% CI – 7, 68) in children aged 3–4 years, and from 26 per 100,000 to 8 per 100,000 (68% reduction, 95% CI 29, 85) in children aged 4–5 years. However, in 2009 the decreases in children aged 2–3 years and 4–5 years were smaller (46%, 95% CI 27, 60 and 11%, 95% CI – 58, 50, respectively) and hospitalizations in children aged 3–4 years increased (–95%, 95% CI −206, −25), suggesting no herd effect. This study had a short period of pre- and post-vaccination observation.

#### Hospitalizations for all-cause gastroenteritis or diarrhea

Five studies on hospitalizations due to gastroenteritis or diarrhea (no confirmation of rotavirus infection) were identified, two in Brazil,^40,44^ two in Panama^39,45^ and one in Mexico.^41^ All the studies showed a reduction in incidence in children aged < 1 year, the age group targeted for vaccination, of 25% (95% CI 14, 34),^40^ 36%,^44^ 18%,^39^ 23%^45^ and 48% (95% CI 46, 50)^41^ (Supplementary Table 5). In Mexico the reduction in hospitalization rate in children aged 0–59 months was 38% (p < 0.001).^41^

**Table 5.** Studies reporting data on potential herd effects against hospitalizations and mortality due to all-cause gastroenteritis and diarrhea after introduction of rotavirus vaccine in Latin America in groups not targeted for vaccination.

References Country	Vaccine type Schedule and age groups Year of vaccine introduction	Coverage and year coverage was assessed	# years before vaccination	# years after vaccination	Subgroups	Relative change in incidence/mortality	Herd protection effect
**Hospitalizations**							
**do Carmo, 2011 [40]** Brazil (national)	Rotavirus vaccine (Rotarix) Vaccination is recommended at 2 and 4 months of age. Vaccine introduction in 2006. The year 2006 was excluded from the analysis. The years 2007–2009 were considered post-vaccination years.	2007^1^ < 1 y: 80%; 1-< 2 yrs: 47%; 2–4 yrs: 0% 2009^1^< 1 y: 84%; 1-< 2 yrs: 81%; 2–4 yrs: 36%	4 (2002–2005)	3 (2007–2009)	**Age groups^2^**		
					1 yr	−21%	Age group not targeted for vaccination ^3^
					2–4 yrs	−7%	Age group not targeted for vaccination ^3^
**Masukawa, 2014 [44]** Brazil (regional)	Oral rotavirus vaccine Vaccination is recommended at 2 and 4 months of age^4^. The oral vaccine of human rotavirus was included in National Program of Immunization (PNI) in March 2006.	2006: 50.13% 2007: 83.51% 2008: 85.81% 2009: 86.61%	6 (2000–2005)	4 (2007–2011)	**Age groups**		
		2010: 91.66%			1 yr	−24,9%	Age group not targeted for vaccination ^3^
		2011: 93.21%			2 yrs	−11,2%	Age group not targeted for vaccination ^3^
					3 yrs	−8,3%	Age group not targeted for vaccination ^3^
					4 yrs	−0,1%	Age group not targeted for vaccination ^3^
**Esparza-Aguilar, 2014 [41]** Mexico (national)	Monovalent RVA vaccine Two doses of the monovalent RVA vaccine – at the ages of 2 and 4 months Vaccine introduction in 2007. 2007 is considered a transitional year and therefore excluded from the analysis.	2010^5^ 0–11 mo: 89% 12–23 mo: 100% 24–59 mo: 69%	4 (2003–2006)	4 (2008–2011)	**Age groups**		
					12–23 mo	−48%	Age group not targeted for vaccination ^3^
					24–59 mo	−18%	Age group not targeted for vaccination ^3^
**Bayard, 2012 [39]** Panama (national)	A two-dose human-attenuated rotavirus vaccine RIX4414; Rotarix A two-dose scheme for children under 6 months Vaccine introduction in March 2006. 2006 was considered to be a transitional year.	2006^6^ 1st dose:62% 2nd dose:30% 2007 1st dose: 89% 2nd dose: 62% 2008 1st dose: 91% 2nd dose: 71%	6 (2000–2005)	2 (2007–2008)	**Age groups**		
					1–4 yrs	−16.5%	Age group not targeted for vaccination ^3^
**Molto, 2011 [45]** Panama (regional^8^)	Monovalent rotavirus vaccine (RV1) Doses recommended at ages 2 and 4 months, and a maximum age of 24 weeks for the second dose Vaccine introduction in 2006. The year 2006 was considered a transition year.	2006 1st dose: 66% 2nd dose:32% 2007 1st dose: 93% 2nd dose: 65% 2008 1st dose: 94% 2nd dose: 72%	3 (2003–2005)	2 (2007–2008)	**Age groups Annual**		
					1–4 yrs	−33,0%	Age group not targeted for vaccination ^9^
					**January-June**		
					1–4 yrs	−45,6%	Age group not targeted for vaccination ^9^
**Mortality**							
**do Carmo, 2011 [40]** Brazil (national)	Rotavirus vaccine (Rotarix) Vaccination is recommended at 2 and 4 months of age Vaccine introduction in 2006. The year 2006 was excluded from the analysis. The years 2007–2009 were considered post-vaccination years	2007^13^ < 1 y: 80% 1-< 2 yrs: 47% 2–4 yrs: 0% 2009^13^ < 1 y: 84% 1-< 2 yrs: 81% 2–4 yrs: 36%	4 (2002–2005)	3 (2007–2009)	**Death rates** **Age groups^14^**		
					2–4 yrs	−4%	Age group not targeted for vaccination ^15^
**Richardson, 2010 [46]** Mexico (national)	Monovalent rotavirus vaccine Recommended at 2 and 4 months of age Vaccine introduction in 2006 and early 2007. 2007 is considered a transitional year.	2008: < 1 yr: 1st dose:74% 2nd dose: 51% 1-< 2 yr: 1st dose: 4% 2nd dose: 2%	4 (2003–2006)	1 (2008)	**Age groups**		
					12–23 mo	−29%	Age group not targeted for vaccination
					24–59 mo	−7%	Age group not targeted for vaccination ^15^
**Bayard, 2012 [39]** Panama (national)	A two-dose human-attenuated rotavirus vaccine RIX4414; Rotarix A two-dose scheme for children under 6 months Vaccine introduction in March 2006. 2006 was considered to be a transitional year.	2006^16^ 1st dose:62% 2nd dose:30% 2007 1st dose: 89% 2nd dose: 62% 2008 1st dose: 91% 2nd dose: 71%	6 (2000–2005)	2 (2007–2008)	**Age groups**		
					1–4 yrs	−28.1%^17^	Age group not targeted for vaccination

mo: months; yr: year; yrs: years; RVA: Species A Rotavirus

Incidence was presented as: hospitalization per 10,000 population individuals (Masukawa et al.) [43], absolute numbers of hospitalizations (Bayard et al.) [38], absolute numbers of diarrhea-associated hospitalizations (Molto et al.) [44].

Change in incidence was calculated by do Carmo et al. [39] (as changes in hospital admission rate per 100,000) and Esparza-Aguilar et al [40] (as change per 10,000 all-cause admissions).

Mortality rate was presented as: death rate per 100,000 individuals (do Carmo et al.) [39], mortality rate per 100,000 individuals (Lanzieri et al.) [42], mortality rate per 100,000 individuals (Bayard et al.) [38]. Change in mortality was calculated by Richardson et al. [45] as relative reduction in rate of death, rate per 100,000 individuals.

1: Completely vaccinated with two doses;

2: Results varied by region;

3: No change in incidence was observed;

4: Not reported in the article of Masukawa [43],taken from the other study in Brazil, do Carmo [39];

5: Completely vaccinated with two doses;

6: In children < 1 year old;

7: The mortality rate did not decline in the first year after vaccination (2006 to 2007). The largest decline was observed from 2007 to 2008, respectively −28% in children < 1 year old and −31% in children 1–4 years old. In 2008 some of the children in the age group 1–4 years old would have received the vaccine; therefore some of the effects might be caused by immunity gained through the vaccine;

8: The goal was to collect data from all 14 health regions, however only six hospitals in five regions fit the inclusion criteria of contributing data for each year of the surveillance and had an average of at least 50 diarrhea-associated hospitalizations annually;

9: In both post-vaccine surveillance years, the decline in incidence of children 1–4 years old is larger than the decline of children < 1 year old.

10: Vaccination schedule not reported. Copied from do Carmo et al. [39];

11: In children < 1 year old;

12: Some of the children in the age group 1–4 years old might have received the vaccine in 2006 and gained immunity, therefore a part of the decline might be caused by the introduction of the vaccine;

13: Completely vaccinated with two doses;

14: Results varied by region;

15: No large change in mortality rate was observed. Small differences might be caused by seasonal fluctuations;

16: In children < 1 year old;

17: In 2007 no reduction was seen in mortality rate compared with mean mortality rate of 2000–2005, however in 2008 mortality rate significantly (p < 0.05) declined in both age groups

Both studies in Brazil showed only a limited decrease in the incidence of hospitalization in age groups not targeted for vaccination (7% [95% CI – 7, 19] in all regions in children aged 2–4 years,^40^ 8.3% in children aged 3 years^44^), indicating little or no herd protection (Table 5). In Panama, both studies reported a decrease in hospitalizations in children aged 1–4 years in the two years after the introduction of the vaccination program (31%^39^ and 40%^45^), which would be consistent with some herd protection. However, by two years after vaccine introduction a proportion of the children in this age group would have received rotavirus vaccination when they were younger, so potential herd effects are difficult to assess. In Mexico, blunting of the seasonal peaks in gastroenteritis in children aged 24–59 months (age groups not targeted for vaccination) was observed in 2011, but not in earlier years. By 2011, almost all children aged < 5 years would have received rotavirus vaccination, so this is likely to be a vaccine effect. The post-vaccination follow-up in these studies varied from 1 to 4 years.

Four studies reported data on mortality due to gastroenteritis or diarrhea, without confirmation of rotavirus infection. Two were conducted in Brazil,^40,43^ one in Mexico^46^ and one in Panama.^39^ All four studies found a reduction in mortality from gastroenteritis or diarrhea in the age groups targeted for vaccination, of 45% (95% CI 40, 51) (p < 0.05),^39^ 22% (95% CI 6, 35),^40^ 39% (95% CI 29, 49)^43^ and 41% (95% CI 36, 47) (p < 0.001)^46^ (Supplementary Table 5). In the study in Panama,^39^ the decrease did not occur until the second year after vaccine introduction, perhaps reflecting low coverage in the introduction year.

One of the Brazilian studies^43^ reported a decrease in mortality from 4.5 per 100,000 in 2004–2005 (pre-vaccination) to 3.0 per 100,000 in 2008 (post-vaccination) in children aged 1–4 years, a decline of 33% (95% CI 15, 52). In view of the low vaccine coverage in this age group (not targeted for vaccination), herd effect could have played a role (Table 5). The other Brazilian study^40^ reported only a small decrease in mortality in children aged 2–4 years (4%; 95% CI – 30, 29), suggesting no herd protection effect. In Mexico,^46^ mortality rates decreased from 21.1 per 100,000 pre-vaccination to 15.0 per 100,000 post-vaccination (reduction of 29%, p < 0.001) in children aged 12–23 months, even though few of these children were of an age eligible for vaccination, suggesting a possible herd effect in this age group. However, there was no significant decrease in children aged 24–59 months (mortality reduction 7%, p = 0.44). The study in Panama^39^ observed a significant reduction in mortality from 20.3 per 100,000 pre-vaccination to 9 per 100,000 in 2008 (p < 0.05) in children aged 1–4 years (not targeted for vaccination), consistent with a herd protection effect (Table 5).

## Discussion

**Figure 2.**
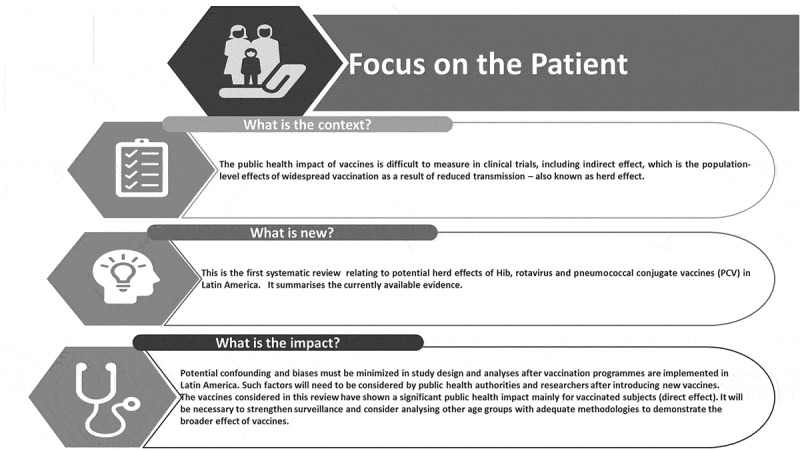
Outcomes and the impact of this study for healthcare providers.

This systematic review covered studies reporting data relevant to herd protection effects after introduction of Hib, PCV or rotavirus vaccination programs in Latin America. A total of 23 studies were identified, five on Hib vaccine, eight on PCV and ten on rotavirus vaccine. All the studies on rotavirus vaccine, all but one of the Hib vaccine studies and four of the eight PCV studies considered only children and/or adolescents. Four studies on PCV^31,33,34,38^ and one on Hib vaccine^27^ covered age ranges including adults. This reflects the epidemiology of diseases targeted for vaccination, which generally have the highest incidence in children, and the focus of the surveillance programs. For some diseases, such as pneumonia, elderly people are also at increased risk. Figure 2 presents a summary of the outcomes and the impact of this study for healthcare providers. Overall, the findings of this systematic review suggest there is currently insufficient robust published evidence to identify and quantify herd effects of rotavirus, Hib or PCV vaccines in Latin America. Robust data may not be obtainable due to the lack of detailed disease surveillance data in non-targeted age groups.

Previous systematic reviews published on herd effects with rotavirus vaccine^25^ or PCV^50^ have not focused specifically on the Latin American region. This systematic review adds to the existing literature by summarizing all available published evidence relevant to assessing herd effects of rotavirus, Hib and PCV vaccines in Latin America. Invasive disease due to pneumococcus and Hib, and rotavirus gastroenteritis, represented a significant public health problem in Latin America before the introduction of vaccination, and it is important to understand the potential indirect effects of vaccination programs beyond the population groups directly targeted for vaccination. To our knowledge, this is the first systematic review of herd protection arising from the Hib, PCV and rotavirus vaccination programs in this region. This review should provide valuable information for decision-makers and may help to indicate potential future research needs.

Few of the studies identified in the present review reported statistically significant reductions in incidence in groups not targeted for vaccination, and the results were not always clear-cut. Methodological limitations may have contributed to these rather unclear results. For instance, several of the studies reported on pneumonia or gastroenteritis episodes in which the pathogen was not specified.^32,39,43,46^ As these diseases can be caused by pathogens other than those targeted by the vaccines, changes in the incidence of other pathogens could affect disease incidence. Furthermore, only one of the studies^34^ had more than 3 years of post-vaccination data. The other studies reported only 1 year^46^ or 2 years^32,39,43,48^ of post-vaccination data, which may have been too short a period to detect clear evidence of herd effect.

Overall, this systematic review suggests there is currently insufficient robust evidence to quantify herd effects in Latin America. This contrasts with results from other regions, where robust evidence of herd effect has been published. Herd effect with rotavirus vaccine has been reported in observational and modelling studies from Europe and North America.^12,14^ Publication bias is always a possibility. Studies with evidence of herd effect might be more likely to be published. It is not likely that publication bias influenced the results of this review. A recent systematic review of herd effect of rotavirus vaccine by Pollard et al.^25^ estimated the median herd effect of 22% against rotavirus-specific gastroenteritis morbidity/mortality (based on 5 studies, 4 of which were from the United States) and 24.9% against all-cause gastroenteritis morbidity/mortality (based on 10 studies, all from Latin America). Herd effect in age groups not targeted for vaccination, such as children in unvaccinated age groups and elderly adults (aged ≥ 50 years or ≥ 65 years) have been reported with Hib and PCV in Scandinavia and North America.^12,14^ A systematic review of herd effect associated with PCV reported that infant vaccination with 3 primary doses plus a booster dose (3 + 1 schedule), 2 primary doses plus a booster dose (2 + 1 schedule), or 3 primary doses with no booster (3 + 0 schedule), has demonstrated evidence of indirect benefits for vaccine-type IPD in age groups not targeted for vaccination in studies from Europe, Australia and North America.^50^ The age groups reported varied between the studies in the review; (further subdivided into different age categories in some studies), others covered the general population including children aged < 18 years and elderly adults aged ≥ 65 years.^50^ Another systematic review found that in countries with mature pediatric PCV vaccination programs, vaccine-type IPD in adults had been nearly eliminated due to indirect protection.^51^ Mature pediatric vaccination programs are defined as those in which high vaccination coverage has been implemented for at least 3–5 years and disease incidence is decreasing.^52^

There are several reasons why robust evidence of herd effect might be lacking in the studies in Latin America and the Caribbean, in contrast with the evidence for herd effect in other regions, such as Europe and North America who have well-established and detailed disease surveillance programs, including laboratory identification of specific pathogens and data analyzed by age groups. Such detailed data are not readily available in Latin America, and this is reflected in the low incidence rates reported in the pre-vaccine era in many cases. This lack of detailed surveillance data in non-targeted age groups limits the ability to detect and quantify herd effect. The authors of a recent review noted that few attempts have been made to estimate herd effects of rotavirus vaccination.^25^ The lack of high-quality baseline surveillance data may make it impractical to conduct a thorough analysis of herd effect of vaccination in Latin America, unless surveillance programs are expanded in the future. PCV was the latest vaccination program to be added in Latin America, and most countries did not include a catch-up program. As a result, the post-vaccination period is currently short. There was some evidence of a possible herd effect in the decreased incidence of overall and vaccine-type IPD in children aged 2–4 years, who were not targeted for vaccination.^34^ In Germany, PCV herd effect was not observed in children aged > 2 years one year after introduction of vaccination, and incidence reductions also lagged in the US.^53^ In addition, many Latin American countries implemented PCV vaccination using a 2 + 1 schedule. In a systematic review of PCV herd effects, the 2 + 1 schedule had evidence of herd effects only against vaccine-type IPD, whereas the 3 + 1 schedule had evidence of herd effects against a wider range of outcomes such as vaccine-type nasopharyngeal carriage and syndromic pneumonia.^50^ Regarding rotavirus, there were important differences between Pollard’s review^25^ and the present review. As well as the difference in regional scope, Pollard et al.^25^ considered herd effect only in children aged < 1 year. This is also the age group targeted for rotavirus vaccination. Pollard et al.^25^ estimated herd effect by calculating the estimated expected vaccine effect and subtracting this from the total observed reduction in gastroenteritis morbidity/mortality. In contrast, the present review sought evidence of herd effect by looking for reductions in gastroenteritis morbidity/mortality in age groups not targeted for vaccination. Furthermore, the analysis conducted by Pollard et al.^25^ combined several outcomes (hospitalizations and mortality) and outcome measures (rates and total event number), whereas in the present review we have reported outcome data directly from the original studies.

Herd effect varies between different diseases and vaccines, and this may also contribute to the lack of a clear picture of herd effect. Herd effect is influenced by many factors, such as vaccine coverage (for primary vaccination and booster doses), catch-up programs, distribution of coverage in the core reservoir of infection, population clustering (e.g. if unvaccinated individuals are likely to be in contact with other unvaccinated individuals), timing of vaccine administration, vaccine effectiveness, the infectiousness of the pathogen, the mechanism of transmission (herd effect is greater in diseases transmitted directly from person to person) and mode of contact (e.g. oral, skin contact, sexual contact).^13,14^ In addition, if the pre-vaccine incidence of disease in age groups not targeted for vaccination was low, it would be difficult to detect a difference after vaccine introduction. Negative indirect effects, such as serotype replacement when non-vaccine serotypes of a pathogen emerge to replace vaccine-type serotypes (as has been documented for PCV-7 pneumococcal serotypes),^14^ further complicate the picture. Thus, it should not be expected that all vaccines will have similar herd effects. Furthermore, extrapolation of herd effect from one geographical area to another, such as extrapolation from studies in Europe to Latin America, should be avoided. This variability also makes herd effect complex to measure. Some studies investigate changes in incidence in the vaccination cohort (e.g. the Pollard review on rotavirus^25^) while others look at unvaccinated age groups (e.g. PCV herd effect in elderly adults). Herd effect is frequently assessed using interrupted time series designs, in which data collected before and after the intervention are compared. Such before-and-after comparison studies of vaccine impact are difficult to interpret,^8^ as they can be confounded by many factors such as changes in incidence, serotype distribution, under-reporting and behavioral factors.^8,16,17^ A review of interrupted time series studies in two systematic reviews concluded that such designs were often underpowered and inappropriately analyzed.^54^ WHO recommendations on the assessment of vaccine impact refer mainly to direct effects, and it may be necessary to define some guidelines for the measurement of herd effect. It would be interesting to study herd effect of vaccines that are available for a long period of time, such as influenza or measles vaccines. One of the methods to evaluate vaccine herd effect for influenza in unvaccinated population would be with a cluster randomized control study.^55^ An interrupted time series analysis could evaluate the impact of measles vaccination in a population once the strategy has been implemented in vaccinated eligible cohorts.^56^

This analysis indicates that the currently available evidence for herd effect with rotavirus, Hib and PCV vaccines in Latin America has a number of limitations. First, most of the studies were of short duration; only 7/23 studies had more than 3 years of post-vaccination data and 12/23 had only 1–2 years of post-vaccination data. This may be insufficient to obtain a full picture of the vaccine impact. Second, several of the studies reported on pneumonia or gastroenteritis episodes in which the pathogen was not specified. As these diseases can be caused by pathogens other than those targeted by the vaccines, changes in the incidence of other pathogens could affect disease incidence. Third, observed changes in disease incidence could also result from factors unrelated to vaccination, such as changes in accessibility of health services, reduced poverty or improved sanitation. Fourth, data on coverage, catch-up programs or booster doses were incomplete or missing from some publications, and information on these aspects would be needed to evaluate herd effects fully. Fifth, no data were available for many of the countries in the region, including Bolivia, Colombia, Costa Rica, Dominican Republic, Ecuador, Guatemala, Guyana, Haiti, Honduras, Jamaica, Paraguay, Peru, Trinidad and Tobago, and Venezuela, and for some outcomes only one study was available (for example, Hib carriage, Hib-confirmed meningitis mortality, incidence of Hib invasive disease, and incidence of pneumococcal pneumonia).

Further research with longer follow-up periods is needed in Latin America to improve the understanding of potential herd protection effects over time and in different population groups, particularly in adults. Similarly, further studies on some of the under-reported outcomes or the countries currently missing from the literature could provide valuable additional information. Additionally, strengthening surveillance for vaccine-preventable diseases before and after vaccine introduction, including groups not targeted for immunization, is also needed to properly understand and identify herd protection.

## Conclusions

This systematic review identified studies reporting possible evidence of herd protection effects with Hib, PCV and rotavirus vaccine against invasive disease, hospitalizations and mortality in children not eligible for vaccination in a range of countries in Latin America. Evidence in adults was limited to only four PCV studies and one Hib study; none of the rotavirus studies included adults. Potential evidence of herd effect was identified for PCV and rotavirus vaccine in children, although the evidence appears less robust than reports of herd effect from other regions such as Europe and North America. This observation may reflect differences in vaccine schedules and methodological limitations such as short post-vaccination follow-up periods (12/23 studies reported only 1–2 years of data after mass vaccination), which may have been too early to detect full effects of pediatric vaccination on adult disease. More research with longer follow-up periods and more detailed surveillance data in different age groups, as well strengthening of the surveillance system in the region would be valuable to measure and report accurately herd effects of these vaccines in Latin America. Although the low pre-vaccine incidence in most cases did not allow quantification of herd protection effects, the remarkably reduced incidences suggest the effects may indeed be there but that larger and longer studies are needed to prove the effects.

## Materials and methods

### Search strategy

The review searched for data on the following outcomes:

- Cases, hospitalizations and deaths associated with gastroenteritis (all-cause or rotavirus-associated);
- Cases, hospitalizations and deaths associated with meningitis, invasive diseases (including sepsis, and all other types of invasive disease) or non-invasive pneumonia associated with Hib or pneumococcus;
- Overall mortality.

The geographical scope of the review covered Latin America and the Caribbean (see Supplementary Text 1 for a detailed list of countries included).

The core searches were conducted in PubMed, supplemented by searches in the Virtual Health Library (VHL; http://bvsalud.org/en/), the Scientific Electronic Library Online (SciELO; http://scielo.org/php/index.phphttp://scielo.org/php/index.php) and SCOPUS. Search strings were developed for rotavirus vaccine, Hib vaccine, pneumococcal vaccine, and Latin America and the Caribbean. For details of the search strings used in each database, see additional file 1. There was no age restriction, except that studies had to include individuals who were not eligible for vaccination, either for full series or catch-up. All age groups were considered in order to maximize the sensitivity of the search. The searches were conducted in 2016.

The searches were limited to articles published from 1990 to 2016, and articles published in English, Spanish or Portuguese. For SCOPUS and SciELO, searches were also limited to the countries listed under ‘Geographical scope’ in additional file 1, as in these databases the use of an additional search string to identify the countries resulted in very few hits.

In addition, a further search was conducted of the grey literature and the following electronic databases:

- World Health Organization (WHO) (http://www.who.int)
- Centers for Disease Control and Prevention (CDC) (http://www.cdc.gov)
- Global Alliance for Vaccines and Immunization (GAVI) (http://www.gavi.org)
- Pan American Health Organization (PAHO) (http://www.paho.org/hq/)
- International Vaccine Institute (IVI) (http://www.ivi.int/web/www/home)
- Google search combining one of the three vaccines with terms for herd protection/immunity and Latin America/South America/Caribbean.

These searches were conducted in 2016. They yielded no additional information.

### Study selection

Publications were screened for inclusion in the review using a three-step process. In the first step, the title and abstract of all hits were reviewed and those which appeared to contain relevant data were selected for full-text screening. Articles were excluded at this step if they were animal studies or cell culture studies, vaccine safety studies, case reports or small case series, modelling studies that did not present original data, excluded publication types (letters, editorials or comments), or if they were in countries outside the scope of this review. If in doubt, the article was retrieved for full-text screening. The first 30% of publications were screened in duplicate by two researchers independently from each other, and the results compared and discussed. The rate of concordance was over 90%. After this, the remaining 70% were screened by a single researcher.

In the second step, the full text of the articles selected at the first step was reviewed. Articles were included if they answered one of the review objectives. Articles were excluded for the following reasons:

- narrative review (e.g. no methods section that described the way the authors collected the literature);
- very poor or insufficient methodological quality;
- efficacy or effectiveness studies without information on herd protection;
- surveillance data describing incidence before vaccine introduction;
- economic evaluation studies without useful information on herd protection;
- very specific populations (e.g. children living in an Indian reservation in Panama);
- article on genotype or serotype distribution without incidence data useful for estimates on herd protection;
- article analyzing the same data as another, already included, article by the same author or study group

The first 10% of publications at the second step were screened in duplicate by two researchers independently from each other, and the results compared and discussed. Different percentages were screened in duplicate between the first step and the second step, because more discussion is often needed to reach consensus at the first screening step (title and abstract) than the second step (full text). Discrepancies were resolved by advice from a third researcher.

Publications could also be excluded during the data extraction process in a third selection step. If several publications presented similar results from the same study, only the most recent publication or that with the most complete study results was included.

During screening, the reference lists of meta-analyses and systematic reviews were checked for potentially relevant articles. No additional publications were identified.

### Data extraction from included articles

Eligible publications were those reporting data for non-targeted populations and/or suggested herd effect and were critically appraised to assess bias following criteria for observational and interventional studies (see additional file 2). We have followed the information in the original reports and have not re-analyzed data from the publications, except to calculate a percentage change in incidence if this was not reported by the original article (see below). Data extraction was performed by a senior researcher and reviewed by the project manager.

If not presented in the original article, the percentage change in incidence was calculated from the mean incidence rate or mean mortality rate before and after vaccination.. A decline in the incidence of cases, hospitalization or death in the population targeted for vaccination is likely to be a direct effect resulting from vaccine-induced immunity, whereas a large decline in incidence or mortality in populations not targeted for vaccination (e.g. age groups not included in the vaccination program) is an indicator for herd protection.

## Trademarks

*Rotarix* is a trademark of GSK group of companies.

## Avaibility of data and materials

All data generated or analysed during this study are included in this published article and its supplementary information files.
