# Supplementary material for: Vaccination herd effect experience in Latin America: a systematic literature review
Source: Hum Vaccin Immunother. 2018 Sep 19;15(1):49–71. doi: 10.1080/21645515.2018.1514225 (PMC6363147; doi:10.1080/21645515.2018.1514225)
Supplement: Supplemental Material [file khvi-15-01-1514225-s001.docx]

**Supplementary Table 1. Studies reporting data on Hib vaccine and possible demonstration of herd effects in Latin America in target vaccination groups and groups with both subjects from the target vaccination group and group not targeted**

| **Reference**  **Country** | **Vaccine type**  **Schedule and age groups**  **Year of vaccine introduction** | **Coverage and year coverage was assessed** | **# years before vaccination** | **# years after vaccination** | **Subgroups** | **Change in incidence** | **Herd protection effect** |
| --- | --- | --- | --- | --- | --- | --- | --- |
| **Hospitalization for Hib-confirmed meningitis** | | |  |  |  |  |  |
| **Miranzi, 2006 [28]**  Brazil (national) | Hib CRM197 conjugated vaccine  Infant <1 year of age  Vaccine introduction in 1999 | 2000: 92% | 16  (1983-1999) | 3  (2000-2002) | **Age groups** |  |  |
|  |  |  |  |  | <1 yr | -80,9%^1^ | Target vaccination group |
|  |  |  |  |  | 1-4 yrs | -80,4%^1^ | Target vaccination group + age group not targeted for vaccination^2^ |
| **Ribeiro, 2007 [30]**  Brazil (regional) | Hib conjugate vaccine  3 doses: at 2, 4 and 6 months of age  Catch up: During the 1st and 2nd year of the Hib immunization campaign, children 12–23 months of age received a single dose of the vaccine  Vaccine introduction in August 1999 | 2000: 82%  2001: 77%  2002: 69%  2003: 86%  2004: 82%  Catch up | 3  (August 1996 – August 1999) | 5  (August 1999 – August 2004) | **Age groups** |  |  |
|  |  | 1999: 37% |  |  | <1 yr | -86,7% | Target vaccination group |
|  |  | 2000: 24% |  |  | 1 yr | -94,7% | Target vaccination group + age group not targeted for vaccination ^3^ |
|  |  |  |  |  | 2 yrs | -88,0% | Target vaccination group + age group not targeted for vaccination ^3^ |
| **Kmetzsch, 2003 [27]**  Brazil (regional) | Conjugate Hib vaccine  The Hib vaccine was implemented for children under 2  Vaccine introduction in 1999. 1999 can be considered a transitional year with low coverage | 2000: 88%  2001: 87% | 1  (1998) | 2  (2000-2001) | **Age groups** |  |  |
|  |  |  |  |  | <1 yr | -77,5% | Target vaccination group |
|  |  |  |  |  | 1-<5 yrs | -83,3% | Target vaccination group + age group not targeted for vaccination |
| **Dickinson Meneses, 2002 [26]**  Cuba (national) | Haemophilus influenzae type b (Hib) vaccine  Infants of 2, 4 and 6 months old were vaccinated  Booster was given at 18 months  Vaccine introduction in 1999. | 1999: 100%  2000: 78% | 1  (1998) | 1  (2000) | **Age groups** |  |  |
|  |  |  |  |  | <1 yr | -70,8% | Target vaccination group |
|  |  |  |  |  | 1-4 yrs | -87,7% | Target vaccination group + age group not targeted for vaccination |
| **Mortality from Hib-confirmed meningitis** | |  |  |  |  |  |  |
| **Miranzi, 2006 [28]**  Brazil (national) | Hib CRM197 conjugated vaccine  Infant <1 year of age  Vaccine introduction in 1999 | 2000: 92% | 16  (1983-1999) | 3  (2000-2002) | **Age groups** |  |  |
|  |  |  |  |  | <1 yr | -82,5%^1^ | Target vaccination group |
|  |  |  |  |  | 1-4 yrs | -81,8%^1^ | Target vaccination group + age group not targeted for vaccination |
| Hib: *Haemophilus influenzae* type b; yr(s): year(s) | | | | | | | |

1: Pre-vaccination mean incidence was calculated using the last five years before vaccine introduction (1995-1999);

2: Some of the children in this age group might have received the vaccine. Change in incidence is likely caused by a combination of vaccine effect and herd protection;

3: During the last year of surveillance no cases of Hib meningitis were observed. The low pre-vaccine incidence does not allow to quantify herd protection effects;

4: Hospitalization incidence rate was very low in adults ≥20 years old. In 1998 incidence was 0 per 100,000 individuals, 0.08 in 1999, 0 in 2000, 0.08 in 2001;

5: Hospitalization incidence rates were already very low before introduction of the vaccine and remained low. The low pre-vaccine incidence does not allow to quantify herd protection effects;

6: Before introduction of the vaccine, the incidence of total bacterial meningitis and bacterial meningitis caused by Hib was already low in children 5-9 and 10-14 years old. After vaccination the Hib meningitis incidence decreased to zero occurrence of Hib meningitis. The low pre-vaccine incidence does not allow to quantify herd protection effects;

7: Mortality rates were already very low in these age groups before introduction of the vaccine. In 2002 no mortality from Hib meningitis was registered in 5-9 year olds and ≥10 year olds. the low pre-vaccine incidence does not allow to quantify herd protection effects;

8: From 1996 to 1999 only one case per year of invasive disease was reported, so although the large decline could indicate a possible herd effect, this could not be quantified.

**Supplementary Table 2. Studies reporting data on possible herd effects against invasive pneumococcal disease and pneumococcal pneumonia after introduction of pneumococcal conjugate vaccine in Latin America in target vaccination groups and groups with both subjects from the target vaccination group and group not targeted**

| **References**  **Country** | **Vaccine type**  **Schedule and age groups**  **Year of vaccine introduction** | **Coverage and year coverage was assessed** | | **# years before vaccination** | | **# years after vaccination** | | **Subgroups** | **Change in incidence** | | **Evidence of herd effect** |
| --- | --- | --- | --- | --- | --- | --- | --- | --- | --- | --- | --- |
| **Overall IPD** |  |  | |  | |  | |  |  | |  |
| **Andrade, 2016 [31]**  Brazil (national) | 10-valent pneumococcal vaccine (PHID-CV)  Vaccination at 2, 4, and 6 months plus a booster at 12 to 15 months of age  Catch-up schedule for children aged 7–11 months (two doses plus booster) and 12–23 months (single catch-up dose) during first year of vaccine introduction  Vaccine introduction in 2010, therefore 2010 is considered a transitional year | 2011: 82%  2012: 88%  2013: 92% | | 2  (2008-2009) | | 3  (2011-2013) | | **Age groups**  **PHID-CV types/total cases^1^** |  | |  |
|  |  |  | |  | |  | | 2-11 mo | -57.6% | | Target vaccination group |
|  |  |  | |  | |  | | 12-23 mo | -62.0% | | Target vaccination group |
|  |  |  | |  | |  | | 2-4 yrs | -38.3% | | Target vaccination group (catch up) + age group not targeted for vaccination |
| **dos Santos, 2013 [33], all serotypes data**  Brazil (regional) | PHID-CV  3 doses in the first 6 months, with a booster dose at 12-15 months of age  Vaccine introduction in 2010 | NR | | 4.5  (Jan 2006-  June 2010) | | 2  (July 2010-  Sept 2012 | | **Age groups**  **All serotypes** |  | |  |
|  |  |  | |  | |  | | <2 yrs | -80.4%** | | Target vaccination group |
| **Valenzuela, 2014 [38]**  Chile (national) | PHID-CV  Vaccination started in 2011 with a 4-dose schedule at 2, 4, 6 and 12 months of age.  In 2012 the schedule changed to 3 doses at 2, 4 and 12 months of age.  Vaccine introduction in 2011 | 2011: 55%  2012: 82%^4^ | | 3  (2007-2010) | | 1^5^  (2012) | | **Age groups**  **All serotypes**  <12 mo | -64.8%*** | | Target vaccination group |
| **Garcia Gabarrot, 2014 [34]**  Uruguay (national) | PCV7, PVC13  PCV7 (March 2008) 2+1 schedule (doses administered at 2, 4 and 12 months of age). During the first year, a 2-dose catch-up program was also offered to children up to 2 years old.  PCV13 (March 2010) same 2+1 schedule was used. Children with 1 or 2 doses of PCV7 completed their schedule with PCV13. Additionally, a single dose catch-up of PCV13 was offered to all children born between 2005 and 2008.  Vaccine introduction: PCV7 March 2008 and PCV13 March 2010 | 2010: 96.9–98.9%^7^ | | 5  (2003-2007) | | 4^8^  (2009-2012) | | **Age groups**  **All serotypes** |  | |  |
|  |  |  | |  | |  | | <2 yrs | -66,1%** | | Target vaccination group |
| **Vaccine-type IPD** | |  | |  | |  | |  |  | |  |
| **dos Santos, 2013 [33], PHID-CV serotypes data**  Brazil (regional) | PHID-CV  3 doses in the first 6 months, with a booster dose at 12-15 months of age  Vaccine introduction in 2010 | NR | | 4.5  (Jan 2006-  June 2010) | | 2  (July 2010-  Sept 2012) | | **Age groups**  **PHID-CV serotypes** |  | |  |
|  |  |  | |  | |  | | <2 yrs | -97.3%** | | Target vaccination group |
| **Garcia Gabarrot, 2014 [34]**  Uruguay (national) | PCV7, PVC13  PCV7 (March 2008) 2+1 schedule (doses administered at 2, 4 and 12 months of age). During the first year, a 2 dose catch-up program was also offered to children up to 2 years old.  PCV13 (March 2010) same 2+1 schedule was used. Children with 1 or 2 doses of PCV7 completed their schedule with PCV13. Additionally, a single dose catch-up of PCV13 was offered to all children born between 2005 and 2008.  Vaccine introduction: PCV7 March 2008 and PCV13 March 2010 | 2010: 96.9–98.9%^7^ | | 5  (2003-2007) | | 4^8^  (2009-2012) | | **Age groups**  **PCV7 serotypes** |  | |  |
|  |  |  | |  | |  | | <2 yrs | -91,8%** | | Target vaccination group |
|  |  |  | |  | |  | | 2-4 yrs | -75,2% | | Largely target vaccination group ^9^ |
|  |  |  | |  | |  | | **PCV13 serotypes** |  | |  |
|  |  |  | |  | |  | | <2 yrs | -72,6% | | Target vaccination group |
|  |  |  | |  | |  | | 2-4 yrs | -54,6% | | Largely target vaccination group ^9^ |
| **Pneumococcal pneumonia** | | | | | | | | | | | |
| **Pirez, 2014 [37]**  Uruguay (regional) | 7-valent pneumococcal conjugate vaccine (PCV7)/13-valent PCV (PCV13)  PCV7: 2 + 1 schedule (given at 2, 4 and 12 months of age). Catch-up immunization was offered to children born in 2007 (2 doses, at 15 and 17 months of age)  Uruguay switched to 13-valent PCV (PCV13) with same vaccination schedule in April 2010. Catch-up immunization was offered to children born from January 1, 2005 to April 23, 2009, with a single dose of PCV13.  Vaccine introduction: PCV7 in March 2008, replaced by PCV13 in April 2010 | | National vaccination data demonstrated high compliance with PCV7/13 use: ≥93% of children received 3 doses (cohort 2008 and 2009) and 98% and 95% have been vaccinated with 1 and 2 doses of PCV13, respectively, for cohort 2010 | | 5  (2003-2007) | | 5  (2008-2012) | **P-CAP**  **0-14 yrs** | | -62,8%^12^ | Groups targeted for vaccination and not targeted for vaccination. ^15^ |
| PCV-7: PCV-7-valent; PCV-13: PCV-13-valent; P-CAP: community-acquired pneumonia caused by *S. pneumoniae;* PCV: pneumococcal conjugate vaccine; PHiD-CV: 10-valent pneumococcal vaccine; mo: months; yr: year; yrs: years. | | | | | | | | | | | |

Incidence in studies presented as: per 1,000 population individuals (dos Santos et al.[32]), per 100,000 individuals population (Garcia Gabarrot et al.[33]) and cases per year (Valenzuela et al. [37])

*A significant reduction (p<0.05) in incidence per 1,000 population individuals was observed.

**A significant reduction (p<0.01) in incidence per 1,000 population individuals was observed.

*** Odds ratios for 12 months 2007-2010 vs. 2011 and 2007-2010 vs. 2012, 12-23 months 2007-2010 vs. 2012, 5-64 years 2007-2010 vs. 2011 showed a significant reduction after vaccination.

1: Cases of IPD caused by PHiD-CV serotypes as percentage of total reported cases of invasive pneumococcal disease;

2: Overall number of cases of invasive pneumococcal disease increased in the period after introduction of the vaccine. Incidence of invasive pneumococcal disease caused by PHiD-CV serotypes increased in adults (>18 yrs);

3: Decline in the age group 2-<15 years was not statistically significant. The incidence dropped from 3.35 to 2.52 per 1,000 individuals in all serotypes and 2.81 to 0.97 per 1.,000 individuals in PHiD-CV serotypes, which is larger than can be explained by only environmental factors. However power of the study might be too low to find a significant effect in this age group.

4: According to the PAHO website;

5: Vaccine introduction in 2011, therefore 2011 is considered a transitional year and excluded from analysis;

6: The decrease is small and only borderline significant in the first year after introduction, but not significant in the second year after introduction. In the 24-59 months there is no significant decline in both years. Small declines could also be caused by other factors, such as seasons, changes in health care, etc.;

7: Among new cohorts;

8: Vaccine introduction in 2008, therefore 2008 is considered a transitional year and excluded from analysis;

9: There is a decline in this age group, but only after 2009, which might indicate this decline is caused by immunity gained by immunization in 2008. Between 2008 and 2009 there was an increase in incidence of IPD;

10: The incidence of IPD declined in PCV-7 and PCV-13 types, but increased in not- PCV- types;

11: The incidence of IPD increased in 15-59 year olds and ≥60 year olds.

12: In 2012 seven cases of pneumonia caused by *S. pneumoniae* were registered;

13: In 2011 and 2012 no cases of pneumonia caused by a serotype of *S. pneumoniae* included in PCV7 were registered;

14: In 2012 two cases of pneumonia caused by a serotype of *S. pneumoniae* included in PCV13 were registered;

15: Since children aged 0 to 14 years are included in one group, it is not possible to separate the effect in age groups targeted for vaccination and not targeted for vaccination. In 2011 and 2012 no cases of P-CAP PCV-7 serotypes were observed, suggesting herd protection. Cases caused by PCV-13 decline to two cases in 2012.

**Supplementary Table 3. Studies reporting data on possible herd effects against all-cause pneumonia after introduction of pneumococcal conjugate vaccine in Latin America** **in target vaccination groups and groups with both subjects from the target vaccination group and group not targeted**

| **Reference**  **Country** | **Vaccine type**  **Schedule and age groups**  **Year of vaccine introduction** | **Coverage and year coverage was assessed** | **# years before vaccination** | **# years after vaccination** | **Subgroups** | **Change in incidence** | **Herd protection effect** |
| --- | --- | --- | --- | --- | --- | --- | --- |
| **Gentile, 2015 [35]**  Argentina (regional) | 13-valent pneumococcal conjugate vaccine (PCV13)  A “2 + 1” schedule (one dose at 2 months old, another dose at 4 months old, and a booster dose at 1 year old).  During the first year after introducing the vaccine, children aged between 12 and 24 months old were also immunized with two doses.  Vaccine introduction in January 2012 | 2012  1^st^ dose: 100%  2^nd^ dose: 83%  3^rd^ dose: 48.3%  2013  1^st^ dose: 87.6%  2^nd^ dose: 84.9%  3^rd^ dose: 61.3% | 3  (2003-2005) | 2  (2012-2013) | **Age groups** |  |  |
|  |  |  |  |  | 0-11 mo | -99,9% | Target vaccination group |
|  |  |  |  |  | 12-23 mo | -38,5% | Target vaccination group |
| **Becker-Dreps, 2014 [32]**  Nicaragua (regional) | 13-valent pneumococcal vaccine (PCV-13)  A “3 + 0” dosing schedule, at 2, 4 and 6 months of age.  During the first year of the immunization program, a single catch-up dose was also provided to children aged 12–24 months.  Vaccine introduction in 2010 | 2011: <1 yr: 63%^2^ (range by municipality: 49–71%); 1-<2 yr: 87%^3^ (range by municipality: 68–100%)  2012<1 yr: 97%^2^ (range by municipality: 80–100%); 1-<2 yr: 98%^3^ (range by municipality: 80–100%) | 3  (2008-2010) | 2  (2011-2012) | **Incidence rate ratio**  **Age groups**  **Hospitalization** |  |  |
|  |  |  |  |  | 0-11 mo | 0.67 | Target vaccination group |
|  |  |  |  |  | 12-23 mo | 0.74 | Target vaccination group |
|  |  |  |  |  | **Ambulatory visits for pneumonia** |  |  |
|  |  |  |  |  | 0-11 mo | 0.87 | Target vaccination group |
|  |  |  |  |  | 12-23 mo | 0.84 | Target vaccination group |
| **Hortal, 2014 [36]**  Uruguay (regional) | Pneumococcal conjugate vaccine, 7-valent and 13-valent  PCV7 (2008): a 2+1 dosing schedule (2, 4 and 12 months of age) and a two doses catch-up, was offered to the 2007 cohort at 15 and 17 months of age.  PCV13 (2010): same dosing schedule, and a catch-up was offered to children up to 5 years of age.  Vaccine introduction in 2008, with a replacement of vaccine type in 2010. 2009-2012 were considered post-vaccine follow-up years. | At least one vaccine dose, by the end of 2012, was 97.7% for PCV7 and 99.8% for PCV13 | 5  (2001-2004) | 3  (2009-2012) | **Age groups** |  |  |
|  |  |  |  |  | 0-11 mo | -8,8% | Target vaccination group |
|  |  |  |  |  | 12-23 mo | -37,8% | Target vaccination group |
|  |  |  |  |  | 24-35 mo | -23,7% | Target vaccination group |
|  |  |  |  |  | 36-47 mo | -18,2% | Target vaccination group |
| PCV-7: PCV-7-valent; PCV-13: PCV-13-valent; CI: confidence interval; mo: months; PCV: pneumococcal conjugate vaccine; yr: year; yrs: years | | | | | | | |

Change in incidence calculated for Gentile and Hortal. Gentile [34] presented incidence rate per 100,000 individuals, Hortal [35] presented consolidated pneumonia incidence per 100,000 person-years; Becker-Dreps [31] calculated incidence rate ratios.

1: There is a small decline of incidence in this age group, however it is not significant and in 2013 the incidence increased. There might be a small effect of herd protection, but natural fluctuations in incidence or effects of environmental factors cannot be ruled out;

2: Received all three doses of the vaccine;

3: Received one dose;

4: The incidence rate ratios of 24-to-50-month-old and 5-to-14-year-old children showed there is a significant decline. However there might be effects of natural fluctuations in incidence or environmental factors that cannot be ruled out.

**Supplementary Table 4. Studies reporting data on potential herd effects against hospitalizations for rotavirus gastroenteritis and diarrhea after introduction of rotavirus vaccine in Latin America** **in target vaccination groups and groups with both subjects from the target vaccination group and group not targeted**

| **References**  **Country** | **Vaccine type**  **Schedule and age groups**  **Year of vaccine introduction** | **Coverage and year coverage was assessed** | **# years before vaccination** | **# years after vaccination** | **Subgroups** | **Change in incidence** | **Herd protection effect** |
| --- | --- | --- | --- | --- | --- | --- | --- |
| **Gurgel, 2014 [42]**  Brazil (national) | Monovalent G1P[8] Rotarix vaccine  Two doses to all children <3 months of age  Vaccine introduction at the end of 2006 | 2006: 28.8% of the patients were vaccinated  2012: 86.7% of the patients were vaccinated | 8  (1998-2005) | 6  (2007-2012) | Data were presented in figures.  Age groups were: -<1 year 1-<2 years ≥2 years |  | The vaccine was associated with reduction in the proportion of children attending the hospital, hospitalizations caused by rotavirus, in all age groups |
| **Sáfadi, 2010 [47]**  Brazil (regional) | Rotavirus vaccine (Rotarix)  Two doses of rotavirus vaccine before the age of 12 months  Vaccine introduction in March 2006. In this study the year 2006 was considered a transition year. | 2006: 54%  2007: 78%  2008: 81% | 2  (2004-2005) | 2  (2007-2008) | **Age groups** |  |  |
|  |  |  |  |  | <1 yr | -82.1% | Target vaccination group |
|  |  |  |  |  | 1-<2 yrs | 73.0% | Target vaccination group |
| **Yen, 2011 [48]**  El Salvador (national) | Rotavirus vaccine  Rotavirus vaccination is recommended for administration in 2 doses at 2 and 4 months of age.  Vaccine introduction in 2006. 2007 was considered a transitional year during which rotavirus vaccine was still being introduced. | 2008, first dose  <1 yr: 76%  1-<2 yr: 84%  ≥2 yrs not vaccinated  2009, first dose | 1  (2006) | 2  (2008-2009) | **Age groups** |  |  |
|  |  | <1 yr: 78% |  |  | <1 yr | -81,5% | Target vaccination group |
|  |  | 1-<2 yrs: 89% |  |  | 1-2 yrs | -82,2% | Target vaccination group |
|  |  | 2-<3 yrs: 84% |  |  | 2-3 yrs | -55,3% | Target vaccination group + age group not targeted for vaccination |
| mo: months; yr: year; yrs: years | | | | | | | |

1: There is a decline in incidence during the transition year, however in the two years after introduction (2007-2008) there is a small increase, but post-vaccine incidence was below pre-vaccine incidence;

2: The years after immunization, 2008 and 2009, did not show a similar trend. In 2008 a decline is seen in age groups not vaccinated, which could suggest herd protection effect. However, this effect is not seen in 2009. In 2009 an increase was seen in rotavirus hospitalizations in children 3 to <5 years old, suggesting no herd protection effect in 2009, while the number of cases in other age groups further declined.

**Supplementary Table 5. Studies reporting data on potential herd effects against hospitalizations and mortality due to all-cause gastroenteritis and diarrhea after introduction of rotavirus vaccine in Latin America in target vaccination groups and groups with both subjects from the target vaccination group and group not targeted**

| **References**  **Country** | **Vaccine type**  **Schedule and age groups**  **Year of vaccine introduction** | **Coverage and year coverage was assessed** | **# years before vaccination** | **# years after vaccination** | **Subgroups** | **Relative change in incidence/ mortality** | **Herd protection effect** |
| --- | --- | --- | --- | --- | --- | --- | --- |
| **Hospitalizations** | |  |  |  |  |  |  |
| **do Carmo, 2011 [40]**  Brazil (national) | Rotavirus vaccine (Rotarix)  Vaccination is recommended at 2 and 4 months of age.  Vaccine introduction in 2006. The year 2006 was excluded from the analysis. The years 2007-2009 were considered post-vaccination years. | 2007^1^  <1 y: 80%; 1-<2 yrs: 47%; 2-4 yrs: 0%  2009^1^ <1 y: 84%; 1-<2 yrs: 81%; 2-4 yrs: 36% | 4  (2002-2005) | 3  (2007-2009) | **Age groups^2^** |  |  |
|  |  |  |  |  | <1 yr | -25% | Target vaccination group |
| **Masukawa, 2014 [44]**  Brazil (regional) | Oral rotavirus vaccine  Vaccination is recommended at 2 and 4 months of age^4^.  The oral vaccine of human rotavirus  was included in National Program of Immunization (PNI) in March 2006. | 2006: 50.13%  2007: 83.51%  2008: 85.81%  2009: 86.61% | 6  (2000-2005) | 4  (2007-2011) | **Age groups** |  |  |
|  |  | 2010: 91.66% |  |  | <1 yr | -35,9% | Target vaccination group |
| **Esparza-Aguilar, 2014 [41]**  Mexico (national) | Monovalent RVA vaccine  Two doses of the monovalent RVA vaccine – at the ages of 2 and 4 months  Vaccine introduction in 2007. 2007 is considered a transitional year and therefore excluded from the analysis. | 2010^5^  0-11 mo: 89%  12-23 mo: 100%  24-59 mo: 69% | 4  (2003-2006) | 4  (2008-2011) | **Age groups** |  |  |
|  |  |  |  |  | 0-11 mo | -48% | Target vaccination group |
| **Bayard, 2012 [39]**  Panama (national) | A two-dose human-attenuated rotavirus vaccine RIX4414; Rotarix  A two-dose scheme for children under 6 months  Vaccine introduction in March 2006. 2006 was considered to be a transitional year. | 2006^6^  1^st^ dose:62%  2^nd^ dose:30%  2007  1^st^ dose: 89%  2^nd^ dose: 62%  2008  1^st^ dose: 91%  2^nd^ dose: 71% | 6  (2000-2005) | 2  (2007-2008) | **Age groups** |  |  |
|  |  |  |  |  | <1 yr | -17.7% | Target vaccination group |
| **Molto, 2011 [45]**  Panama (regional^8^) | Monovalent rotavirus vaccine (RV1)  Doses recommended at ages 2 and 4 months, and a maximum age of 24 weeks for the second dose  Vaccine introduction in 2006. The year 2006 was considered a transition year. | 2006  1^st^ dose: 66%  2^nd^ dose:32%  2007  1^st^ dose: 93%  2^nd^ dose: 65%  2008  1^st^ dose: 94%  2^nd^ dose: 72% | 3  (2003-2005) | 2  (2007-2008) | **Age groups Annual** |  |  |
|  |  |  |  |  | <1 yr | -22,9% | Target vaccination group |
|  |  |  |  |  | **January-June** |  |  |
|  |  |  |  |  | <1 yr | -45,7% | Target vaccination group |
| **Mortality** |  |  |  |  |  |  |  |
| **Lanzieri, 2011 [43]**  Brazil (national) | Rotavirus vaccine  Vaccination is recommended at 2 and 4 months of age^10^.  The vaccine was introduced in 2006, therefore this year which was therefore considered a transitional year. | 2006^11^  1^st^ dose: 60%  2^nd^ dose: 39%  2007^11^  1^st^ dose: 85%  2^nd^ dose: 72%  2008^11^  1^st^ dose: 77%  2^nd^ dose: 90% | 2  (2004-2005) | 2  (2007-2008) | **Age groups** |  |  |
|  |  |  |  |  | <1 yr | -34.5% | Target vaccination group |
|  |  | In 2008, an estimated 38% of the population 1–4 years of age had received one vaccine dose, with 28% having received two doses. |  |  | 1-4 yrs | -31.1% | Target vaccination group + age group not targeted for vaccination ^12^ |
| **do Carmo, 2011 [40]**  Brazil (national) | Rotavirus vaccine (Rotarix)  Vaccination is recommended at 2 and 4 months of age  Vaccine introduction in 2006. The year 2006 was excluded from the analysis. The years 2007-2009 were considered post-vaccination years | 2007^13^  <1 y: 80%  1-<2 yrs: 47%  2-4 yrs: 0%  2009^13^  <1 y: 84%  1-<2 yrs: 81%  2-4 yrs: 36% | 4  (2002-2005) | 3  (2007-2009) | **Death rates**  **Age groups^14^** |  |  |
|  |  |  |  |  | <1 yr | -22% | Target vaccination group |
|  |  |  |  |  | 1 yr | -28% | Target vaccination group |
| **Richardson, 2010 [46]**  Mexico (national) | Monovalent rotavirus vaccine  Recommended at 2 and 4 months of age  Vaccine introduction in 2006 and early 2007. 2007 is considered a transitional year. | 2008:  <1 yr:  1^st^ dose:74%  2^nd^ dose: 51%  1-<2 yr:  1^st^ dose: 4%  2^nd^ dose: 2% | 4  (2003-2006) | 1  (2008) | **Age groups** |  |  |
|  |  |  |  |  | 0-11 mo | -41% | Target vaccination group |
| **Bayard, 2012 [39]**  Panama (national) | A two-dose human-attenuated rotavirus vaccine RIX4414; Rotarix  A two-dose scheme for children under 6 months  Vaccine introduction in March 2006. 2006 was considered to be a transitional year. | 2006^16^  1^st^ dose:62%  2^nd^ dose:30%  2007  1^st^ dose: 89%  2^nd^ dose: 62%  2008  1^st^ dose: 91%  2^nd^ dose: 71% | 6  (2000-2005) | 2  (2007-2008) | **Age groups** |  |  |
|  |  |  |  |  | <1 yr | -19.9%^17^ | Target vaccination group |
| mo: months; yr: year; yrs: years; RVA : Species A Rotavirus | | | | | | | |

Incidence was presented as: hospitalization per 10,000 population individuals (Masukawa et al.) [43], absolute numbers of hospitalizations (Bayard et al.) [38], absolute numbers of diarrhea-associated hospitalizations (Molto et al.) [44].

Change in incidence was calculated by do Carmo et al. [39] (as changes in hospital admission rate per 100,000) and Esparza-Aguilar et al [40] (as change per 10,000 all-cause admissions).

Mortality rate was presented as: death rate per 100,000 individuals (do Carmo et al.) [39], mortality rate per 100,000 individuals (Lanzieri et al.) [42], mortality rate per 100,000 individuals (Bayard et al.) [38]. Change in mortality was calculated by Richardson et al. [45] as relative reduction in rate of death, rate per 100,000 individuals.

1: Completely vaccinated with two doses;

2: Results varied by region;

3: No change in incidence was observed;

4: Not reported in the article of Masukawa [43],taken from the other study in Brazil, do Carmo [39];

5: Completely vaccinated with two doses;

6: In children <1 year old;

7: The mortality rate did not decline in the first year after vaccination (2006 to 2007). The largest decline was observed from 2007 to 2008, respectively -28% in children <1 year old and -31% in children 1-4 years old. In 2008 some of the children in the age group 1-4 years old would have received the vaccine; therefore some of the effects might be caused by immunity gained through the vaccine;

8: The goal was to collect data from all 14 health regions, however only six hospitals in five regions fit the inclusion criteria of contributing data for each year of the surveillance and had an average of at least 50 diarrhea-associated hospitalizations annually;

9: In both post-vaccine surveillance years, the decline in incidence of children 1-4 years old is larger than the decline of children <1 year old.

10: Vaccination schedule not reported. Copied from do Carmo et al. [39];

11: In children <1 year old;

12: Some of the children in the age group 1-4 years old might have received the vaccine in 2006 and gained immunity, therefore a part of the decline might be caused by the introduction of the vaccine;

13: Completely vaccinated with two doses;

14: Results varied by region;

15: No large change in mortality rate was observed. Small differences might be caused by seasonal fluctuations;

16: In children <1 year old;

17: In 2007 no reduction was seen in mortality rate compared with mean mortality rate of 2000-2005, however in 2008 mortality rate significantly (p<0.05) declined in both age groups
